# Supplementary material for: Marine origin of retroviruses in the early Palaeozoic Era
Source: Nat Commun. 2017 Jan 10;8:13954. doi: 10.1038/ncomms13954 (PMC5512871; doi:10.1038/ncomms13954)
Supplement: Supplementary Information — Supplementary Figures, Supplementary Tables, Supplementary Notes and Supplementary References [file ncomms13954-s1.pdf]

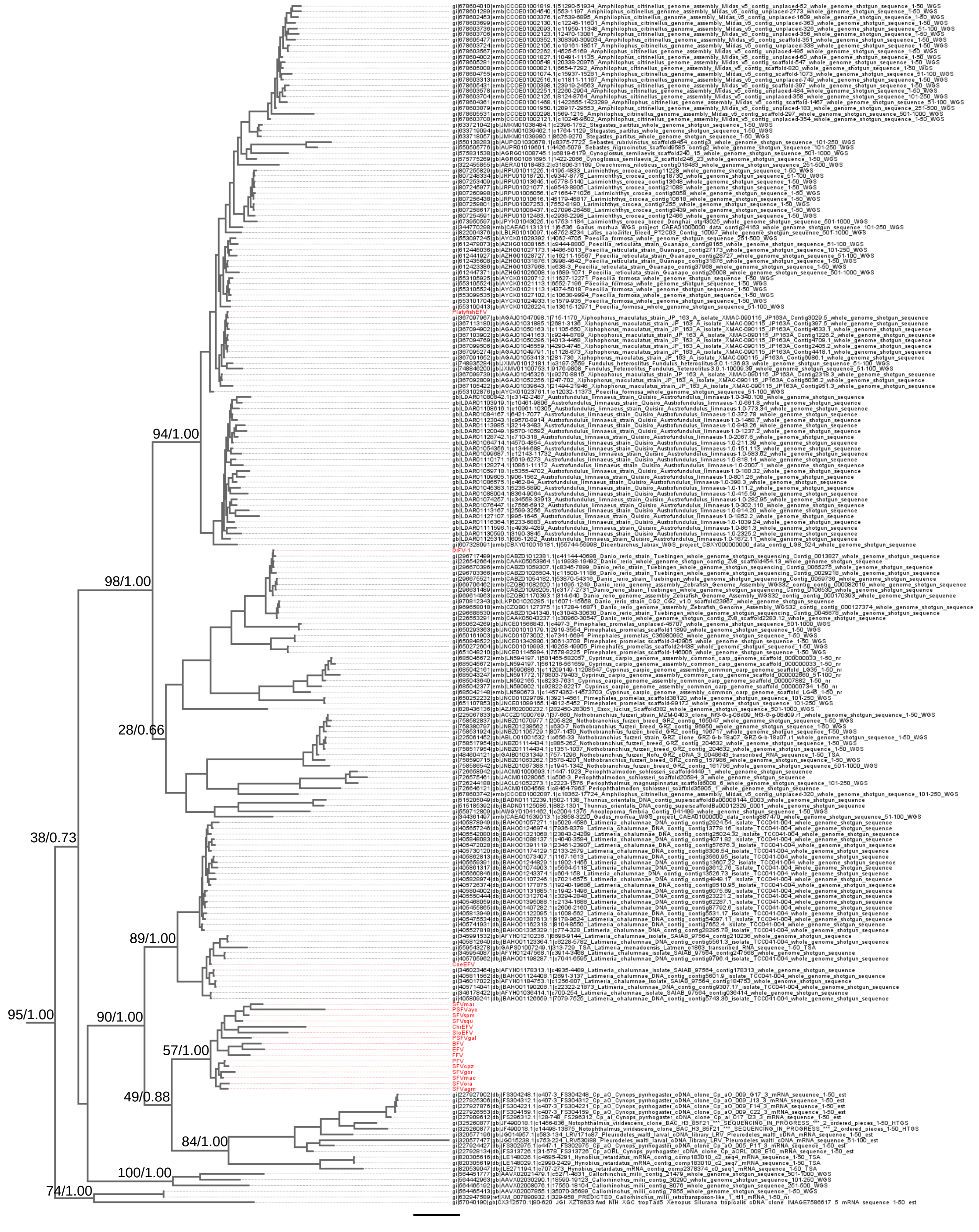

Supplementary Figure 1 Phylogenetic relationship among FVs and FLERVs inferred from reverse transcriptase protein sequences. The scale bar (black solid line, underneath the tree) shows genetic divergence of length 0.2 in units of amino-acid substitutions per site. Since both Bayesian maximum clade credibility and maximum likelihood trees have very similar topologies, only the Bayesian tree is shown. The numbers on nodes are bootstrap (1st) and Bayesian posterior probability (2nd) clade support values. Known mammalian FVs and fish FLERVs are labelled in red.

Supplementary Figure 2

NviFLERV-1 (JF490018: c17,626-8,434)

5′-LTR

1 **TG**TCACATGCTTTTTGCAAAAGCCTTGCTTTTTTGCTTAGGTTGACCTGACTTTATAATTTTCCACCAGGCCTGTTTTAG 80

81 TTGCACACGGCCCTTAGCTGAGGCTCGGTTTGTTTGGCGCTTGCCTCTGCCCTAGATACATATGTGCAGACATGTTGTTT 160

161 CCCTTCACTCCTGCCCTAGTACAGTGCAAGGCCATTTAGTACGCTGAGATCGGCCATAGACAGATTTCTAGAAATAAACA 240

241 GGCAGTGCAGYTTTTCCCATGCAAAGAAGCCCCTTATCACTAGGTGTTAGGCGCGAACAAGGCAGAACGACTTTGCTCTT 320

321 CTCTGGTTCAAGATGCTGCTGCCAAAGCCTGCAGGCCTGATCCATMTCGCACACATGACAAAWACYTAGACTCAGGTATG 400

401 GGTTYGACCACACAGAGACATTTCCAACTTTACTCTTTAGTCTAAGTTCTGGGCAGGACTACTTTGTCTAACTATATTTT 480

481 AGACACTATACTCATTTTACTGTGATTTCTAGCATAATATAACATGTTAGTCATATTATACTTTTATATTTGTGCATTAG 560

561 CAATTGTATTATTTCTCTCTGTGATATGCATGGTAGTTGCAGTACAACAGATTTGTAGACTGAGATCATAGTCGTATAAT 640

641 AAAACCTATATAATTACATCTCCGTCTCTTTATCATTATTTTGAGTGCATATAAGTCTCCAAACCGAAAAAGTGATCTTT 720

721 ACTTGCAAGACCACTTTCCCATGAGATCGGGAGGGGAGTCCTCTGTTCTAGGTTGCCCATATACTTGCTGAGGGTTGTTG 800

801 CTGTATAATTTTTACTAAGAATAAGAAGGCAAGTAGGGCACTGTAGCTAGCCGGACAAATTTGGGGTTTCTGTACTGATT 880

881 GGTCAAGAACGCATTTAGTGGACACAAGAAGTACACAATTCCCTTAGGCACAGTTTTGTACCCACAGAAACCATGCAA**CA** 960

PBS

961 TGGCA**TACCCAACGTGG**TGGGGCTCTTATCTGCAGGTACACCTGAGTAGTACTTATATGCATGCATAATACCCTTACGCC 1040

1041 TTTAATATAGGAGAAGTCCTGGGTGTTTGGAATAATTCCTCCGCGGTGTTCAGGCTAAACCGAACTCCGTGGTTTTTCAA 1120

1121 TGCATTGAAGTTTAAAAGGAAAATAAACCCAATATTGTGTTGCCTTTAAATTCTATTATTCTACATTCACATTACACATA 1200

Gag ORF

1201 CACTAATTCTTTTTATAGAAATTAGCGTTTAAAAACTATCGTTTTTTTGTAATTCAAAATGGCGGTGGCAGCAGCTGTTA 1280

M A V A A A V N

1281 ACATACAGCCCGCCGTTAGACAAGCTCTTTTCACTCACCTGACTAACCACCGTTTTACACCCCCTGGTGGTGTCATGATC 1360

I Q P A V R Q A L F T H L T N H R F T P P G G V M I

1361 TTTGCAGTCGAAGCTAGCGATCAATATCGCTGCAAATTTTATTACTCATGGGTGGAGTTTCCGGCTGTT--ACCACAAAG 1440

F A V E A S D Q Y R C K F Y Y S W V E F P A V ? P Q R

1441 ATACGCTCCATATCTATGGCTTGAAGTACTGTTATCAGTGTGGAATCATAACGCGTGGTTTGGTGTTCAATTACCCCATA 1520

Y A P Y L W L E V L L S V W N H N A W F G V Q L P H T

1521 CTCTAAGGCCGATAAGACTAGGACCTTTAAATAATCCTGGTGCAACATGGCCCACTCTCGCTGGTTTTACACCCTATGTC 1600

L R P I R L G P L N N P G A T W P T L A G F T P Y V

1601 GATACTGCAAATAGAACGCGAGGTGAAATGGTAGCATTGTATGAACAAGTCTGTCAGATGTATTGGATCTTGGTGCAATA 1680

D T A N R T R G E M V A L Y E Q V C Q M Y W I L V Q Y

1681 TATGACACGGACTTTTATCAGTGCTCCTGGACTGCCAGGTCAGCCACAAGCACCGGCCCACGGCCAAAGCATTGGACCCG 1760

M T R T F I S A P G L P G Q P Q A P A H G Q S I G P V

1761 TGGTTGTAAATTCTCTTATGGGCAAAAAGCCCGTCACCAGGGAGGAATATCCCTACTGGTTAGCAGAGAAAATACCGCAA 1840

```
     V   V   N   S   L   M   G   K   K   P   V   T   R   E   E   Y   P   Y   W   L   A   E   K   I   P   Q
1841 TTGGAAGCGGTATTACCACAATGTAATCCTCAAGACAGGCACTGTATACTAACGATGTGCCTGCCCCAAGGAATTGTTCC 1920
     L   E   A   V   L   P   Q   C   N   P   Q   D   R   H   C   I   L   T   M   C   L   P   Q   G   I   V   P
1921 TACACAAGCTGAATGGAGCGAGGCATTTGCGGCTTTGTACATTATCGCTCACGGGGAACCATCCTTTGCAGTGTTGCCAG 2000
     T   Q   A   E   W   S   E   A   F   A   A   L   Y   I   I   A   H   G   E   P   S   F   A   V   L   P   E
2001 AGGTTTTGAAACAAATTCAAAACCAACACGGAGCAGCTGCAGCCCTAGATTTAGGGATGCGCCCTCTGGCAAACTTTCCC 2080
     V   L   K   Q   I   Q   N   Q   H   G   A   A   A   A   L   D   L   G   M   R   P   L   A   N   F   P
2081 GTGGTTTCCCAGATGATTTTGAGAAACATTAAGGACGAAGCTGCGGCCTTGATCTTGCGCCAACAACTGATGGCGTTACC 2160
     V   V   S   Q   M   I   L   R   N   I   K   D   E   A   A   A   L   I   L   R   Q   Q   L   M   A   L   P
2161 GGTTATGCAAAGAGAGGTGGCTTTACCCGGTATAATTGCCGATACCTATCAGGCGATGGGACGTAACAATTTAGGTAACT 2240
     V   M   Q   R   E   V   A   L   P   G   I   I   A   D   T   Y   Q   A   M   G   R   N   N   L   G   N   C
2241 GTCCGCCCAAAACTCTGCCGGAACAACCTAAAGAGAAGCCGGCTGGAAAGCCCCATGACACTTCTAAAAAGACTTGGGTA 2320
     P   P   K   T   L   P   E   Q   P   K   E   K   P   A   G   K   P   H   D   T   S   K   K   T   W   V
2321 CAGACTAAAAATAAGCAGAACAAACAGGGGAAGAAAACTGAGCAATCTCAGTCCTCTGAGAGATCTGAAAAACAGTATGA 2400
     Q   T   K   N   K   Q   N   K   Q   G   K   K   T   E   Q   S   Q   S   S   E   R   S   E   K   Q   Y   E
2401 ACTGCGCGACCGCTCTAAAGTGAAGCCTCCAGACAGGTATCAGGGTACTGATACTCGTTCTTCTCGTTCCCCACAGGAAC 2480
     L   R   D   R   S   K   V   K   P   P   D   R   Y   Q   G   T   D   T   R   S   S   R   S   P   Q   E   P
2481 CGTCCACTTCACAGGGAGGCCCACAGCGATCAGAAGGCAGATACGACGAACAAACGCGAAGGCGCTCTGGTCTTGACTAA 2560
     S   T   S   Q   G   G   P   Q   R   S   E   G   R   Y   D   E   Q   T   R   R   R   S   G   L   D   *
2561 GACCAAAAAGTCAAGCAAGAACAGCAAAGTCCAAAGCAGAAACCGCAATTTCAATCAAAGAAAGTAGCTGCATTGACTGA 2640
     D   Q   K   V   K   Q   E   Q   Q   S   P   K   Q   K   P   Q   F   Q   S   K   K   V   A   A   L   T   E
               Pol ORF (uncertain stating site)
2641 ACACTACGCCTCAAGTACTGAGGACACTGATAGCGGGGAACCGCTGGGCACTGGCGGTTCTGGACAGCGCGGCAGAGATT 2720
     H   Y   A   S   S   T   E   D   T   D   S   G   E   P   L   G   T   G   G   S   G   Q   R   G   R   D   Y
         T   P   Q   V   L   R   T   L   I   A   G   N   R   W   A   L   A   V   L   D   S   A   A   E   I
                                             Gag ORF end
2721 ACCGTTATACTTTGGAATCTACAGGACATTCTGGCAGCGACTTCAACTGAACAGTTTGTCGCGGTTGAAACTGCAGATAC 2800
     R   Y   T   L   E   S   T   G   H   S   G   S   D   F   N   *
     T   V   I   L   W   N   L   Q   D   I   L   A   A   T   S   T   E   Q   F   V   A   V   E   T   A   D   T
2801 ACATGTATTACCACCAGACAGGATTTATAATGTCAAAATCCAAATTGAGGGTGATATTATACGTTCCATCCAAGCTATTT 2880
     H   V   L   P   P   D   R   I   Y   N   V   K   I   Q   I   E   G   D   I   I   R   S   I   Q   A   I   F
2881 TTTGGGACAATTTTACATCTACTTATGACATTCTTTTAGCTGAGGCAGACTGGCCTCCGGACTTTGTACGCTCCGCACCA 2960
     W   D   N   F   T   S   T   Y   D   I   L   L   A   E   A   D   W   P   P   D   F   V   R   S   A   P
2961 TGTGGTGAGGACGTCATTCTCCCTGCTTTTTCTTCGCTTGTCCCTGACAATGTTAAATA-GCTTATGCGGCTGATTGGGC 3040
     C   G   E   D   V   I   L   P   A   F   S   S   L   V   P   D   N   V   K   ?   A   Y   A   A   D   W   A
```

3041 TTTGGCACTGGCCCCAGCACTCTATCGCAATGAATGCGGCTGGGATGAAGAATCACCTTCTCACGTAATCCCTAAACGTA 3120

L A L A P A L Y R N E C G W D E E S P S H V I P K R T

3121 CTAGCCCGCAAGTGCAACCTCAATATCCTATCAAACGTGAAGCAAAAGCTTCGGTCAAGGAAATACTCACTCATCTTGAG 3200

S P Q V Q P Q Y P I K R E A K A S V K E I L T H L E

3201 AATCAGGATGTCATTGAATCATGTACTTCAGATATGAACAATCCTCTTTTTCCTGTGGCCAAACCTGACCATTCATATCA 3280

N Q D V I E S C T S D M N N P L F P V A K P D H S Y H

3281 CATAGTCTTAGATTATAGACACCTGAACAGTCACACACGTTCATTTGCGATCCACAATACACACAGTTCAACTCTTGTAA 3360

I V L D Y R H L N S H T R S F A I H N T H S S T L V N

3361 ACAATCTGACTCGAAAAAAATACAAAACCACGTTGGGCATTTCCAACTGATTTTTCACACAAAATCTAGCACCTGAAAGC 3440

Reverse transcriptase domain

N L T R K K Y K T T L G I S N * F F T Q N L A P E S

3441 CAAGACTGGTCCACATTTACCTGTCTCGGTATCCAATACAAATTTTGGCGTCTCCCAATGGGGTATAAGAATAGCCTGGG 3520

Q D W S T F T C L G I Q Y K F W R L P M G Y K N S L G

3521 AGTGTTTGCGGTGCGTATTATCGAATTACTGCAGTTAGTAGACCCTGACGCGGTGTCGTACATTGACTATATTTACGTCA 3600

V F A V R I I E L L Q L V D P D A V S Y I D Y I Y V T

3601 CGGACGATGAAATGCAACAACATTTGGCCCGGGTGGATCGCATAATCATACATTTTTCTGCCTCCGGCTACAAAATAAAC 3680

D D E M Q Q H L A R V D R I I I H F S A S G Y K I N

3681 TTCAAAAAATCTAAAATTGCTTTCTTTAGTGTCCTGTTTCTGGGATACAAACTCAACAACGAGGGCAAGAGCTTAGCTCC 3760

F K K S K I A F F S V L F L G Y K L N N E G K S L A P

3761 TCATTTTCTTGAGCGCTGCGCAGTGCTTCAGGATCCAAACACGCTTACAAAACTTCATTCTTTGTTGAACTTTCTCAACT 3840

H F L E R C A V L Q D P N T L T K L H S L L N F L N F

3841 TTGGACGTAGATACATACCAGAATATGCACAGAGAATAAAACCTCTCTACAGTTTGATTGAGCATGACTACTGTTTAACA 3920

G R R Y I P E Y A Q R I K P L Y S L I E H D Y C L T

3921 CACTGGACTGATGCGCACACTGACATTCTCCGTTCCCTGCAACGAGATTTGCTGGAACTTTAACACTTACACACACGTGA 4000

H W T D A H T D I L R S L Q R D L L E L * H L H T R D

4001 CAATACCACAAACCTTGAGATCCGCGTCTCACCAAGCGCGACCGGATTCACTTACATGACTTTCAATGAAGGGGACACGG 4080

N T T N L E I R V S P S A T G F T Y M T F N E G D T V

4081 TCCCCATGGCATATAAATCGCATTTATATTCATCTGTTGAGATGCGTTTTGCCTCTACTGATTTTTTTTTTACTGCGGTG 4160

P M A Y K S H L Y S S V E M R F A S T D F F F T A V

4161 CAGATGGCGATCCTCAAGGAACGCCCGCTTGCACAAGGAAAACGCATTATTGTGGTGACACCGGTACCAGCCCTCGAAGC 4240

Q M A I L K E R P L A Q G K R I I V V T P V P A L E A

4241 TGTCACCAAGGCTAGTGTCCCAAATGCAAAAGCCCTGCATCCGAGATGGATACAGTGGGCAGCGTCACTGACTTCAGTTG 4320

V T K A S V P N A K A L H P R W I Q W A A S L T S V D

4321 ACGTAGACTTTGTCTTTGACACTACACTACAATCTCAAGAATTCTTGCAATACGAAGAAATCTATCCGCAACGTCCTTCG 4400

V D F V F D T T L Q S Q E F L Q Y E E I Y P Q R P S

4401 GTTTTGCCTCTTGATCAGTATCAACAAATCATTTACATTGACGGGAGTGCTCAACCGGCCATTGGTTCAAAGCAGGCGTA 4480

V L P L D Q Y Q Q I I Y I D G S A Q P A I G S K Q A Y

4481 TTCAGCGGCTTGTTCTACAATCACTGGCGTAATGAAGGATGGTGTCTTTCATCCTACAAAAACACGTACAATGTCTCTAG 4560

S A A C S T I T G V M K D G V F H P T K T R T M S L G

4561 GAGACTGTACTACACAGCTGGCTGAGCTCACATCTTTAGTCCTTGCACTTACACATGCAAAACAAGACATTCTGACACTC 4640

D C T T Q L A E L T S L V L A L T H A K Q D I L T L

4641 ATCATTTCAGACTCTCACTATTGTGTTCGCGCCTATA-TGAGGACCTACATTACTGGCGTTTGAATAATTACAGGGATTC 4720

RNaseH domain

I I S D S H Y C V R A Y ? E D L H Y W R L N N Y R D S

4721 GAAGGGCAACATAATAAAGCACAAAACGCTTTGGGTACAGGTGGCTACCTTTAAAGGCCAGCTACCGCAGACTCATGTAG 4800

K G N I I K H K T L W V Q V A T F K G Q L P Q T H V V

4801 TACATACATTGGGTCATCAACGTGTGGGCTCGCACGTTGTAGGAAATACTTTAGCAGCTACTGCGGCCAAGTCTGCAGTT 4880

H T L G H Q R V G S H V V G N T L A A T A A K S A V

4881 TCTCTGGGTACTGTTGCTGCAATTACACGTTCTCAAACGAAGATAGACGCTGATATCTTGGCGGCTGTCCATGCATCTCA 4960

S L G T V A A I T R S Q T K I D A D I L A A V H A S Q

4961 GGAAGGAATCCCTCCCCCAAAAGGCTTTCCCATTAAATATACATATTGCTTAAGCTATCAAAACATTGCTTTTGTACAAA 5040

E G I P P P K G F P I K Y T Y C L S Y Q N I A F V Q I

5041 TACCTGGGGTTGGAGATAGAACGCTCCCCAATCAAGATCGCAGATTAGCTTTAATTAAGGCAGCCCACGAAGGTGTGGCT 5120

P G V G D R T L P N Q D R R L A L I K A A H E G V A

5121 TCTGCTCATGTGGGTGTCCAGGCTACCATCACTTTGCTACAGCAGCAATACTGGTGGAAAGGGCTGTACAAAGAAGCTAG 5200

S A H V G V Q A T I T L L Q Q Q Y W W K G L Y K E A R

5201 GAAATATGTTCTCGAATGTGACATTTGCCAGCAAATTAAAACCTCCACCATAGTCAGACCACCTCAAACTTCACTCTTCA 5280

K Y V L E C D I C Q Q I K T S T I V R P P Q T S L F I

5281 TTTCAGATAAACCCTTTGGTTGTGCTTACCTTGACCGTTGTGGCCCTTTGTCTTCTGATACTGCATACAAGTATATCTTG 5360

S D K P F G C A Y L D R C G P L S S D T A Y K Y I L

5361 GTCGCAGTGGATTCTTGTTCAAGATTCCTCTGGGTGTGGCCACAGCGCTCGGCTGACGCTCGTACAGTTATTAAAGATTT 5440

V A V D S C S R F L W V W P Q R S A D A R T V I K D L

5441 GCGAGTTTTATC-GGAACGTATGCATGTCGTACATTCCATGCAGACCAAGGCCCGGCATTCGCTTCTAAAGCGTACCGGG 5520

Integrase core domain

R V L ? G T Y A C R T F H A D Q G P A F A S K A Y R E

cPPT

5521 AGGCTCTAGAGTCCCTGGGGGTACAAATACATTACAGCTCACCCTACAGACCCGAGGGTAATAGTGTAGTGGAGAGAATG 5600

A L E S L G V Q I H Y S S P Y R P E G N S V V E R M

5601 AACCGTGACTTAAAGCAATCCTTAACGTTGCGAGTTATAGGTATGGGACGGGACTGGCTGCACCACCTGTATGGGGTCCA 5680

N R D L K Q S L T L R V I G M G R D W L H H L Y G V Q

5681 GATGGGTCTGAACAACTTGCCCAGACAGTCTCTGTGTGGCCGAACTCCTTATGATGTCTTATTCGGCCAATCCATGTTTG 5760

M G L N N L P R Q S L C G R T P Y D V L F G Q S M F V

5761 TGCCAGACCTTGACGGTCTTACTGTTGTGTCGGCGGACACATCATATGATCTCGATAACAGATTCGTGCTGACGTCACTG 5840

P D L D G L T V V S A D T S Y D L D N R F V L T S L

5841 GCG-CCCAGTCTGAAAAGAAACCCGCTGCTGCTGGTGAAACTGCTCCTTCCATTCCTGTGGGCTGGCTTCCGAAGAAAGG 5920

A ? Q S E K K P A A A G E T A P S I P V G W L P K K G

5921 AGACCTGGTCCGTGAGAAGATTGCAGCTAAGAAGGAGTTTGGACCATCCTATCGTTGTCCAGTACCAGTTCTGGGGATAT 6000

D L V R E K I A A K K E F G P S Y R C P V P V L G I S

6001 CCGGAACTCGAACGGTGATCCTGCCACCACTTCCAGGTTCGACTGATCCCCGTTTTGTTTCTTCCCATCCTGGGGGGGTT 6080

G T R T V I L P P L P G S T D P R F V S S H P G G V

6081 ACTACTTACTCTATTGGTAAATTATGTCAAAAATGGCTGAAAGATTCCACTTCATCAGCAGTGAAAGACCATATCGCAAC 6160

T T Y S I G K L C Q K W L K D S T S S A V K D H I A T

Pol ORF end **Env ORF (uncertain starting site)**

6161 GTTGTCTGATAC-TAGTGATTTTAATGACATTATACTTGGTCCACGTACACCACGTTCTAAACGTTTTGCTTCCATTGCT 6240

L S D ? *

L I ? S D F N D I I L G P R T P R S K R F A S I A

6241 TATATAATTTACAATGAAATATCAAAGGTTTCTAAATTAGATGCTGCAATACGTTTACATCAAATTGATGAAGACAACAT 6320

Y I I Y N E I S K V S K L D A A I R L H Q I D E D N M

6321 GAATAAAGTTTTAGGTGTGGTTGATAATAGTATCAACACACTGTCTTCACGTATTTATGATTTGGAAAATGTATTTTCTT 6400

N K V L G V V D N S I N T L S S R I Y D L E N V F S S

6401 CTGTTATTGATATTGTGCAGTCTGATGTATCCACATTTTATCATGTCCAGACACAATTGAGGCCCATTTTAGATTTCAGT 6480

V I D I V Q S D V S T F Y H V Q T Q L R P I L D F S

6481 TGGACCCTTCAAACTCTGAAAAGTGGTAAAGTCCCGTGGTCATACTTAAATGTTTCATCATTGTTTTCACGTTTTAACTT 6560

W T L Q T L K S G K V P W S Y L N V S S L F S R F N L

6561 GACACGCTCACAACGATTAATGGCTCAAAAGAGTGCCACTTATACTATGTTACATTTAGAAAAGTTAGAACGTTTACCTT 6640

T R S Q R L M A Q K S A T Y T M L H L E K L E R L P F

6641 TTACTGTCTCTGAAATGTCCTCCACAGTCTGGTTAATCCACGGGGTTATTACTCTTCCCATTGCCACATTTAAATTCACG 6720

T V S E M S S T V W L I H G V I T L P I A T F K F T

6721 GACTGTATGAAATACATTCCCTTCAGGTCCTGCACGAAGATAGCTGACTCCTACATTCATGAAAAATGGTTTCTTCCTTT 6800

D C M K Y I P F R S C T K I A D S Y I H E K W F L P F

6801 CCTATACAGATGCTACAGTGGAGAGAAAGAGGTTTTTCTCTCAGTGCGGGACTGCGACTCCTCTTTGGGGCACTCGCTTG 6880

L Y R C Y S G E K E V F L S V R D C D S S L G H S L V

6881 TCTGCCAAGAAATCACAGTTCACGGTGCATGTACTGCTTCCACACCAAATTTGTCCTGCGCTTTGCGTGGGCTTCCAGTT 6960

C Q E I T V H G A C T A S T P N L S C A L R G L P V

6961 TCCTTTATTGCAGGCAAAACTCATGTTTTAGTGAATGGCAGTTATGTTATGCTGAACGAAATGGATTGCTGTGGTATGAT 7040

S F I A G K T H V L V N G S Y V M L N E M D C C G M I

```
7041 AGCTGGAATTCCGTATATTGTCACAGTTTCCCATCCGGTAACTTGTTGCGGAGTCCTTATCTTTCCTCCCTCCCACAAGA 7120
      A  G  I  P  Y  I  V  T  V  S  H  P  V  T  C  C  G  V  L  I  F  P  P  S  H  K  T
7121 CGCTGGAAAGCTCTATTTGGCCTCACATTCCAACTCAGAATTACAATTTTGATAAGCTGAGCAGGCTCAAAGCGTTACTG 7200
       L  E  S  S  I  W  P  H  I  P  T  Q  N  Y  N  F  D  K  L  S  R  L  K  A  L  L
7201 TTTCATAAAAAAGTTTACTTGACATCTGCTTGTGAGACCTTCAACTTGCAGGTGGCGACAACTTCCAGTGAAATACAGTC 7280
     F  H  K  K  V  Y  L  T  S  A  C  E  T  F  N  L  Q  V  A  T  T  S  S  E  I  Q  S
7281 GCTGTTGAACACTAATCTCCCTATACATTTTTGTGAACTCCTTCATACTATTTCTAATGCTTCGTCCAAGACCGGCCTTG 7360
      L  L  N  T  N  L  P  I  H  F  C  E  L  L  H  T  I  S  N  A  S  S  K  T  G  L  V
7361 TACACGTTTTTAAGGTGGTGGACTCGGGCGTGGTTTCGACCTTTAAAGCAGTGGTAGGTATTATACCAGCTGCTATCCAT 7440
       H  V  F  K  V  V  D  S  G  V  V  S  T  F  K  A  V  V  G  I  I  P  A  A  I  H
7441 TCCCTATTTTCGAGTTTCTTTGGGTTTGTCCCAATACTTCTCTGCGCTATAGCTGCAGTGGTGGTTCTGTTCCTTTTTCT 7520
     S  L  F  S  S  F  F  G  F  V  P  I  L  L  C  A  I  A  A  V  V  V  L  F  L  F  L
                    Env ORF end                        Acc ORF (uncertain starting site)
7521 CCGTTATGGATGCCCGACTCGGAAACGTCCTGCGTCAGGCCTTGCTCATGAAATGCCGCCAGTGACTGCTTGATCTGCTT 7600
      R  Y  G  C  P  T  R  K  R  P  A  S  G  L  A  H  E  M  P  P  V  T  A  *
                                                                      L  L  D  L  L
7601 GAGATAGCAGTTGTGCTGGACCTTGGCCAACACTGGTCTCTGACATTCCGCTCTCAATGGTACAACTGTATCATTCCGGT 7680
     E  I  A  V  V  L  D  L  G  Q  H  W  S  L  T  F  R  S  Q  W  Y  N  C  I  I  P  V
7681 CTTACGTTGCCTCTGGTGTCCCTTTCAATTGCTGATTGATGTCTGCTTGGCTCAACATCTGTCTTTTCTTATGGACTTGG 7760
      L  R  C  L  W  C  P  F  Q  L  L  I  D  V  C  L  A  Q  H  L  S  F  L  M  D  L  E
7761 AGTTACTAATGGACCCACTGAGGGCCCATTGTCACGGGTGTCCACGTCGATTAAGATTGATTCAAGAGTCTACAATTGAG 7840
       L  L  M  D  P  L  R  A  H  C  H  G  C  P  R  R  L  R  L  I  Q  E  S  T  I  E
7841 CTTCCCTCGTTTCCTATCTCAGGTGCGGACGCACAGGAAATGAGCTCTGCGGACATCCATGCCACGTTGACTGGCATCTT 7920
     L  P  S  F  P  I  S  G  A  D  A  Q  E  M  S  S  A  D  I  H  A  T  L  T  G  I  L
7921 GGATGTTCTCGTTCCGCTCTTAGATTCGAGCTGTCACAACTGCTACCTACCGCTGCTCAACTCTGTTCTCTCTGCTATGC 8000
      D  V  L  V  P  L  L  D  S  S  C  H  N  C  Y  L  P  L  L  N  S  V  L  S  A  M  Q
                                                                 Acc ORF end
8001 AGCCCGTGGATGCTGCTTCTTCAACTGGTAACCTGGATCCTTTGGAAGTCTTTCTGGGAACCCTTGACACTCTCTAATCT 8080
       P  V  D  A  A  S  S  T  G  N  L  D  P  L  E  V  F  L  G  T  L  D  T  L  *
8081 GAACTTTTTGACAAAATTGTCCCATTTTTTCTTAACGTGACGTCTTTTTCATTTTTTACTATTTCGACCTATTTTTATTA 8160
                                                                    PPT       3'-LTR
8161 TGATCATTAGCTTCTGAGCTAATATATGTCTCTTCTCTCCTGGGTCTACAACCGATCTTAGGGGGGTGACACATGCTTTT 8240
8241 TGCAAAAACCTTGCTTTTCTGCTTAGGTTGGCCTGACTTTATAATTTTCCACCAGGCCCGTTTTAATTGCAGACGGCCCT 8320
8321 TATTTGAGGCTCGGTTTGTTTGGCGCTTGCCTCTGCCCTAGATACATATGTGCAGACATGTTGTTTCCCTTCACTCCTGC 8400
8401 CCTAGTACAGTGCAAGGCCATTTAGTACGCTGAGATCGGCCATAGACAGGTTTCTAGACATAACACGGCAGTGCAGCTTT 8480
8481 TCCCATGCAAAGAAGCCCCTTACCACTAGGTGTTAGGCGCGAACAAGGCAGAATGACTTTGCTCTTCTCCGGTGCTAGAC 8560
```

8561 AGACGATCCTCGATGCTGCTGCCAAAGCCTGCAGGCCTGATCCATCTCGCACACATGACAAAAACCTAGACTCAGGTATG 8640

8641 GGTTTGACCACACAGAGACATTTCCAACTTTACTCTTTAGTCTAAGTTCTAGGCAGGACTACTTTGTCTAACTATATTTT 8720

8721 AGACACTATACTCATTTTATTGTGATTTCTAGCATATTATAACATGTTAGTCATATTATCCTTTTATGTTTGTGCATTAG 8800

8801 CGATTGTATTATTTCTCTCTGTGATATGCATGGTAGTTGCAGTACAACAGATTCGTAGACTGAGATCATAGTCGTATAAT 8880

8881 AAAACCTATATAACTATATCTCCGTCTCTTTATCATTATTGTGAGTGCATATAAGTCTCCAAACCGAAAGGGTGATCTTT 8960

8961 ACTTGCAAGACCACTTTCCCATGAGATCGGGAGGGGAGTCCTCTGTTCTAGGTTGCCCATATAATTGCTGAGGATTGTTG 9040

9041 CTGTATAATTTTTTACTAAGAATAAGAAGGCAAGAAGGGCACTGTAGCTAGCCGGACAAATTTGGGGGTTTTGTACTGAT 9120

9121 TGGTCTGGAACGCATTTCGTGGACACAAGAAGTACACAATTCCCCAGGCACAGTTTTGTACCCACAGAAACCACGCAA**CA** 9200

NviFLERV-2 (JF490018: c4,391-1)

5′-truncated 5′-LTR

1 TGCTGCCCCAGCTGCTAGGCCTGATCACCATTGCACGAATGCAAAACCCCTAATAACCAGGTGAGGAGTTCTGTGCACAA 80

81 CAATGACATTTCCTACTTTATAATTTCTAGCTTAGTCCTTGGCAAGTTTTACTTTAGCCTAGCTATAAAATAGGCACTAT 160

161 AATTGTTTACTGACAGATATACAGTAGCAGTGTTATACAATAATATGGTGGTAACACTAGCTTTCTTTGTATGTGTATTT 240

241 TCAGTCATTATCTTTCTGGCTGGTATTTGCGTGGTAATCTCTATGCAACAGATCCGTAGAGCTAGACAATATGTACTATA 320

321 ATAAAACACTATATTGCTATTTCTAAGACTCTGTGTTACTTATTATTACGAGTGTGTATGAGTTCTAATTCTCTAGTCTA 400

401 ACACGAAAAGGTGATTACTGCTTGCAAAGACCACGTTTCCTGAGACCGAAAGGGAGTCCTCTGTTCTAAGTACGCCCAGA 480

481 CACTTGTCGAGTGTTCATTCCTATAATTATTACTATAACCAAAAAGACAAGTGGGGTACTGTAGCTAGCTGGACAAAGTG 560

561 GGTTTGCGGTCACCGATTGGTAAGGTTACGCATTTCCCTATTACAAAAAGTACGCAAACCCCAGCACAGTTCTGAACCTG 640

PBS

641 TCAGAAACTCTGTAA**CATGGCGCCCCAA**CCGTGATGGGTCTCTTATCCCGCAAGTACACCTGAGTAGTACTCGTATGCAC 720

721 TCATAATACCTTTACGCCGTATATTGGAGACGTCCTCTGGTGTTTAGGAAATAATTAATTAATTCCTCCGCGGTGTTCAG 800

801 GCTGCGACCGAACACGCGGTTTTTCGATGCGCATTGAAGTTTAAAAAGGAAACCCACTTTTGTATTGCCTTTTGGAATCT 880

881 ATAGATTTACCTATTATAGTCTTTTCATACGTTTGTTACACTATTTAGACATATATTGTACCACACACGAACTTACATTT 960

Gag ORF

961 CTAACTGCGACACATTTTCTGAAAAACAAAATGGTGGACGCGGAAGAAAACGAAGAAGCCGTGGCTATTCACGCGGCAGT 1040

M V D A E E N E E A V A I H A A V

1041 TAGAGAAGCGGTTCACACACACTTACTACGCCATCACTTCACAGACGCTGGTGGTATATTAATTTTTGTTGTGGAAGCTA 1120

R E A V H T H L L R H H F T D A G G I L I F V V E A S

1121 GCGCTGAAAATCCCATGGAATACTTTTATTCCTGGGTGGAGTTCCCGGCAGTCGATCAAGTTACGGGACATTTCCACACT 1200

A E N P M E Y F Y S W V E F P A V D Q V T G H F H T

1201 TACACAATTCCAGCCCTTCCTAATGTTTACGTTCCCTATCAACGTGTCGAGGTACCGTTAACATACTGGAACCACCGAAC 1280

Y T I P A L P N V Y V P Y Q R V E V P L T Y W N H R T

1281 GTGGTTTGGGAGCGAAATTCCGCACCCCGCCGAGATTGTTCGACTCGGACCTTTGGGTGTGCAGGGGGGGGCTCATTGGC 1360

W F G S E I P H P A E I V R L G P L G V Q G G A H W P

1361 CAGCGCTTGCAAACTACACTCCTTATACTGAGTATAATGACCTCGATAAAAATGACGTAATTAATCTTTACGAACAGATC 1440

A L A N Y T P Y T E Y N D L D K N D V I N L Y E Q I

1441 ACCGGGCTTTACCGGACTTTGGTGCGCTTTACATCGCGCACTTTGGTTGCAGTACCGGTTCAGCCTGGTGTTCCACATAT 1520

T G L Y R T L V R F T S R T L V A V P V Q P G V P H I

1521 CGTTCAGCCACAACACGGGCAGATAATTGGACCCGTGGTGGTGAACGCACTCATGGGTAAAAAACCGACAGTACGGGACG 1600

V Q P Q H G Q I I G P V V V N A L M G K K P T V R D D

1601 ACTATCCCTTCTGGCTGGCTGAAAAAATACCACAGCTAGAAGGCGTCTTTACCCATTCCACCCCACAGGACAGGCACCGT 1680

Y P F W L A E K I P Q L E G V F T H S T P Q D R H R

1681 ATTTTAACCATATGCCTGCCCCAAGGTATGGTTCCCACGATTCGTGAATGCAATTCTTGGAGTGAAGCGTTCGCAGCACT 1760

I L T I C L P Q G M V P T I R E C N S W S E A F A A L

1761 GTACGTCATTGCTGTGGGTTCGCCCTCCATAGGTGCCATTCCTGAAGTCTTAAAACAAATACAGGGCATTCATGGTGCAG 1840

Y V I A V G S P S I G A I P E V L K Q I Q G I H G A A

1841 CCGCAGCCCTGGATTTAGGCATGCGCCTGATGGGCAACTTTGAGATGATTTCACATATAGTCATTCAAAATATCAAAGGC 1920

A A L D L G M R L M G N F E M I S H I V I Q N I K G

1921 GAAGCTGCAGCATTAATGGTAGAAAGCCGTATGATGGCTTTGCCTGTACAAGAAAGAGAAGCGGCATTACCAGGTATTAT 2000

E A A A L M V E S R M M A L P V Q E R E A A L P G I I

2001 TGCTCGGGTCTATAAGACACTGGGTCGTAATAATTTAGGTAATAAGCCACTCAAACCCACCGACTTGGCAAAAGACAAGC 2080

A R V Y K T L G R N N L G N K P L K P T D L A K D K Q

2081 AGGTAACAAAACCCCAGGAGACATTTAAAAAGTCCTGGGTGCAAAATAAACAACCAAAACAAAAGAAGGAAACGCGGACG 2160

V T K P Q E T F K K S W V Q N K Q P K Q K K E T R T

2161 AGGGAGAGTTCCCCGTCCACGGATTCCACCGGACGCAGGTATGAATTACGTGATCGCTCTAAAATAAAATCACCAGACAG 2240

R E S S P S T D S T G R R Y E L R D R S K I K S P D R

2241 ATTTCAGGTTCCTGACACACCTCGTTCTTCTCGTTCCACACAGGACTCATCGTCCTCTAGAGAAGGATGGAAACCTGATC 2320

F Q V P D T P R S S R S T Q D S S S S R E G W K P D R

2321 GCAAACACAAGATCGTATTGCGAAGGCTAGCTACAACGGCTTCTGACTCCGACCGAAAAGTCAAGAAGGAGCCGCAAAAA 2400

K H K I V L R R L A T T A S D S D R K V K K E P Q K

3'-truncated Pol ORF

2401 CAATTCAAGACAAAGACAACGAAAGTAGCTGCATTGTCTGCCCACCATGCCTCAAGTGTTACGGGTTCAGCTGGCGGACA 2480

Q F K T K T T K V A A L S A H H A S S V T G S A G G H

M P Q V L R V Q L A D I

2481 TCCAATGGGCACTGGCTCTCCTAGACAGCGCCGCAGAGATCACGATCATACGCAAGACTTTGTTGCATCGTCTGAAAGCG 2560

P M G T G S P R Q R R R D H D H T Q D F V A S S E S D

Q W A L A L L D S A A E I T I I R K T L L H R L K A

Gag ORF end

2561 ACAGCAACTGATCGCTTTGTATCAGTCGAGACAGCAGACACACATGTATCACCACCCGACAGAATATATTCTGTGGCACT 2640

S N *

T A T D R F V S V E T A D T H V S P P D R I Y S V A L

2641 TCAAATTGAGGGTGATCTTCGACGTCTCATAGATGCTATATTTTGGGACAAGCTAACGTCTACTTATGACATTCTTCTAT 2720

Q I E G D L R R L I D A I F W D K L T S T Y D I L L S

2721 CTGAGGCAGACTGGCCTCCGGACTTTATCAGATCCGCTCCACGCGGTGAAGAAATCATACTTCCTGCTTTTTCTTCACAT 2800

E A D W P P D F I R S A P R G E E I I L P A F S S H

2801 GTTCCTGATGAACTTAAACATTCATATGCAGCATCTTGGGCTTTGGCACAAGCACCCGCGTTATATCGCAACCAGTGCGG 2880

V P D E L K H S Y A A S W A L A Q A P A L Y R N Q C G

2881 CTGGGATGAGAAATCTGATCCTCATGTCATTCCCATACGTACTGAACCACATGTGCAGCAGCAATATCCTATCAAAAAGG 2960

W D E K S D P H V I P I R T E P H V Q Q Q Y P I K K E

2961 AAGCGAAGGCTTCGGTTAAGGAAATACTCAAACAACTTGAGTATCAGGGTGTCATTGAACAGAGCAAATCTGAAATGAAC 3040

A K A S V K E I L K Q L E Y Q G V I E Q S K S E M N

3041 AATCCACTTTTTCCTGTAGTTAAACCTGATAATACATATCGCATAGTCTTAGATTATCGCCACCTTAACAGTTGCACGCG 3120

N P L F P V V K P D N T Y R I V L D Y R H L N S C T R

3121 TTCATTTGCGATACAAAACACACACAGTTCAGGACTTGTGAATAATTTGACACGAAAAAAATGGAAGACCACTTTGGACA 3200

S F A I Q N T H S S G L V N N L T R K K W K T T L D I

3201 TTTCCAATGGATTCCATGCTCAGAATCTGGCGGAAGAAAGCCAGGACTATTCTAGTTTTACCGGAATTGGCATCCAATTC 3280

S N G F H A Q N L A E E S Q D Y S S F T G I G I Q F

3281 AAATTTCGGCGACTCCCAATGGGGTACAAGAACAGTCCTGGAATATTCGCAGCACGAATTATAAACATACTGCAAAAAAT 3360

Reverse transcriptase domain

K F R R L P M G Y K N S P G I F A A R I I N I L Q K I

3361 TGACCCCGATGCAGTGTCGTATGTTGATGACATCTACGTCACGGACGATGAAATCCGCATACATTTAGCCCGGGTGAATA 3440

D P D A V S Y V D D I Y V T D D E I R I H L A R V N K

3441 AAATAATTCTCTGCTTTGCAGCCCATGGCTATAAAATAAACTTTAAAAAATCTAAAATTGCTTTTTTCAGTGTCCTGTTT 3520

I I L C F A A H G Y K I N F K K S K I A F F S V L F

3521 CTGGGATACGAACTCCATGATGAGGGCAAAAGCCTTGCGCCTCATTTTCTTGAACGTTGTGCGGTGCTTCAAGTGCCAAA 3600

L G Y E L H D E G K S L A P H F L E R C A V L Q V P N

3601 CACGCTCACAAAGCTTCATTCTCTTTTAGGCTTTCTTAATTTCGGACGCACTTACGTTCCAAACTACGCACAGAGAATAA 3680

T L T K L H S L L G F L N F G R T Y V P N Y A Q R I K

3681 AACCTCTCTATGAACTGATTGCACCAGACTACAGTTTAAAACACTGGACTGACGCACACACTGACATTTTACGTTCTTTA 3760

P L Y E L I A P D Y S L K H W T D A H T D I L R S L

3761 CAACAAGACCTGTTAGAACAAAAATATCTACACACACGTGACAATACCATGAACCTTGTGATCCGTGTTACACCCAGTGC 3840

Q Q D L L E Q K Y L H T R D N T M N L V I R V T P S A

3841 AACCGGGTTCACCTATTTGACATTCAATGAGGGGGACACAGTCCCGGTGGCATATAAATCGCATTTATATTCACCAGCTG 3920

T G F T Y L T F N E G D T V P V A Y K S H L Y S P A E

3921 AAGTCCGTTTTGCCTCCACTGAAAAAATACTGACGGCTGTGCAAATGGCGATTCTTAAAGAACGCCCTTTGGCACAAGGA 4000

V R F A S T E K I L T A V Q M A I L K E R P L A Q G

4001 AAARGAATTATTGTGGTGACACCTGTGCCGGCGCTGGAAGCCGTCACTAAAGCGAGTGTACCGAATTCAAAGGCACTACA 4080

K ? I I V V T P V P A L E A V T K A S V P N S K A L H

4081 CCCTAGATGGATACAGTGGGCAAGCTCGCTTACCTCTGTTGACGTCGACTTTGTTTTTGACCCTACACTTCAAACACAAG 4160

P R W I Q W A S S L T S V D V D F V F D P T L Q T Q E

4161 AATTCTTACAATACGAGGAAATCTACCCATTGCCTCGTGAGGTTGTGCCTCTCGATCAGTATCACAAAGTTATCTATACT 4240

F L Q Y E E I Y P L P R E V V P L D Q Y H K V I Y T

4241 GACGGAAGTGCCCAACCGGCCATAGGGAGCAAGCAAGCATACAGCGCTGCCTGTGCCACTGTCACTGGTACCATCACTGA 4320

3’truncated RNaseH domain

D G S A Q P A I G S K Q A Y S A A C A T V T G T I T D

4321 CGGTGTCTTTACTCCCACAAAAACACGTACTATTTCTTTAGGGGATTGTACAGCACAATTGGCTGAGCTTA

G V F T P T K T R T I S L G D C T A Q L A E L ?

AciFLERV

5′-LTR

1 **TG**TCGAATGAAGTGGGTTGAGGTTGAGATCTTGGTTATTCGATGGCCTGTGCCTGGTACTATGTGGACAATCATAATTCT 80

81 GATTTATGTAAAATGATAAAGTTGATTAATGGAATGTAACAAATAATCTGTATGATTTCTAATCAAAGTTGAGAAGAGTG 160

161 TGTGTAACACAGAAATGATTTGAGCACAGCATGTAAAGGAAGTTGTGAAATCTTGGAGAATCACTGAGCAGGGTAAAATG 240

241 AGGAACTGAAACCTGTTGCCTAGGAATCAGAAGGAACAGAAGGGAAAACAAGTTAAGAATAGTCTTAACTGGAAAAGTAC 320

321 CAAACAAGCCATGTATTACAAGAAATGAAGTAATGGTCAAAGCCAAATACCATAATTGGTAATCAAACAGTGGAGTGATA 400

401 ATTTGTCATGATGATGTTTTAAAACTAGGATGTGTCTTAGACTTAAACTATAAAAGGCGATGCACAGAAGCATCGGGTGA 480

481 GATTCTAGGGAGCCCTTCCCAGCACCAGGACGAGTCTACAGATTGGACCAAGCCAACCCAGATGTGGACATTTAATTGAC 560

561 TTTAAACGTCTTTGTGAGAATTCTCCCTGCATTGTCAGATGAGGATTGGAAGTACCTAAGCTGTTGGATTTCAAAGAGGA 640

641 TTTCCGAAGAACAGTAAGGATTATAAAAGAAGGACAGTCAACTGAAGGGAGTCAACATCTCTCTTTTTGGACTTCTCCTT 720

721 TTTCCCCCCAACTTCACTGAGGCAGTAACGGACTGGACTTTGGGTGTGGTCGCTTCGGCCGAGTGACTCACTTGAAATTC 800

801 AGTCTCTTCAAAAAGGATTTATGGACAGAATTGGACATTTTCAAGCCCCTTCTTCATCAACCCAGCTCATAAGTAACCAT 880

881 TTTGATTGAGATTAAGTTTTTGATTTATTCTGTTTTTGAAGTTTAGATTTTGAGCTGATTGTTGACATTCCAGAATTGAT 960

961 TGAGAATTGATTTTGAGCTAGATGCAAACTGCTTGAGCTGAAAACCTGCATAATAAAATTTGACTCTAAAAAGTCAGTAT 1040

1041 AATTCGTTGAGAATTAATTCCCTTTTTAACGGTACACTTATGTCCATGCAACAGTAAAACTCTAGGCAAGTTAAGCTATA 1120

1121 ACATCTGGTTCTATTTTGGACACACTAGTCGTAAGTAATCCACTTCAACTTAACTTATGTCCCTTGGGCTCACTGTCCGA 1200

1201 GCCGATAAAAGAAATCTGGTCGGTAACAAGCGCACTGATCAAAGGGGCTGGCTGCTATAACTATCTGGGCTGTATTTTGG 1280

1281 GGTCGGCTGCGTGAGCCTTCCTGACCGGCAGCATCAGAGTACAGAGGAAGCGGGGCAGATGTTAGGATTATTAGCAGTTA 1360

1361 GAGCCAGACGAGCAGCCCCGCCACAGCTTAAGTAGTGTCACTGAGCTCAGAAGTGGGTAATACCCTGCTGGCCAAGTGAT 1440

PBS

1441 CAGGACCCTATGAAACGGAGTAGCTGTCACAGAGGTAAAATACCTGTGGGATCTAA**CA**CG**TGGCGCCCAACGTGGGGC**GT 1520

1521 GGCCGAAAAAAGAGGATTTCGCCGCGCCTAAAGAACCTAGATAGCCCGGAGAGGTTATCATTCGTGGGAGATAACCCCAG 1600

1601 GATTGATTGAAGGAGCAGCGCGAGGCAGAGAGGACTTCGTGAGCTGTTCAGGAGCGTAAGCGGGGATCAACCGACGCTCT 1680

1681 CAACCTGTCCAGGAAGCGGAGTGACGCGGACAGGAGTAGCAGCAGACGTCGCTGAGGACACCCAGACCGACAGGGGCCCA 1760

1761 GAGGACTCTGCAGGAAGGCAACGCTCGACTCCATCCCTTGCCTCGGAAGTACAACGAGGGGCCTTGGAGGAGAGCAACTC 1840

Gag ORF

1841 GAGACGACAAAGACAAAGAGCGCCGGACCAGTCTAGAGATGGCTGAGGCGACTGATGAAGGAGTGCCAGAGGAGCCCCAA 1920

M A E A T D E G V P E E P Q

1921 CCAGAAGGGGTGTCCCTCTTTAAGTCACTCAAGGCACCCGGCGAGGAGGAACCGGAGGCGCCTGCCCCATCGGAGGCAGG 2000

P E G V S L F K S L K A P G E E E P E A P A P S E A G

2001 GTCGACGGAGGCGCAGGGAGCAACAGGGCCGGCAGTATCCCAGGTCCAGGCATGGGCGATGATTCATGTTGTCCCATGGG 2080

S T E A Q G A T G P A V S Q V Q A W A M I H V V P W G

2081 GACAAGGATGGAAGAAATGGGAAGCTGCAATCCAGCATCACGTAGCTTCAAGGTGTATCGACATGGATGAAGGAGAAGTT 2160

Q G W K K W E A A I Q H H V A S R C I D M D E G E V

2161 GGTAAGGCGGTGGATGCAGCTTGCAGCAAAAACCAAGCTTATCGGACACCCGAAGTGTTGAACCGAATTTGGGTTCAAGT 2240

G K A V D A A C S K N Q A Y R T P E V L N R I W V Q V

2241 GGAACCGTCCCCTGAGCCCGGGACGAAGAAGGATATAATACAGAAGTGGCTGGAATGGGCAGTAAAGGTAATATGGCCCG 2320

E P S P E P G T K K D I I Q K W L E W A V K V I W P D

2321 ACGGGGTAGAGGAAGAACCGCGACCCATAGCATCGTGGATTAGTCCCCAACCAGAGTATGATCCTATTATTAAGATGTTG 2400

G V E E E P R P I A S W I S P Q P E Y D P I I K M L

2401 GGAAAGGTGACAGGTACTGGTGCAGTTGAAGTAGAAACAAAAGTTACTGCAGCTAAGCAGTACAAAGAACACCGAGAGTT 2480

G K V T G T G A V E V E T K V T A A K Q Y K E H R E L

2481 ATTGTTACAGTTGGAAAAGTATCAGGGGAGGGCTGAGGAACTCTTCAAGGACCCCCCTGAAAGTATAGTGGGATCCCAGA 2560

L L Q L E K Y Q G R A E E L F K D P P E S I V G S Q M

2561 TGGAGGATGGAGAAACCGGCTCGGAGGATGACTCTGAATTGGATTCTGAGAGAGATGGAAAATGGGATCTAAAGGGAACA 2640

E D G E T G S E D D S E L D S E R D G K W D L K G T

2641 ACACTGCACCTAGCGGACCTGCCCTACAAGCTTGAGAGTGCATCAGAGACACCACGGAAGGTGCAACCCCTCCTGCGGGA 2720

T L H L A D L P Y K L E S A S E T P R K V Q P L L R E

2721 GCAGGAGGACCGGCTGAGGGAGAAGTATAAATGGACTTCCCCATTTGGCCTGGTGGACGAACCAGTTCGAGAGGAGACTC 2800

Q E D R L R E K Y K W T S P F G L V D E P V R E E T P

2801 CGTCGGACTGGCTCCCTAAACTTGATCCTGTCCCAGCGGAGGCCCTGAAGTGGAGGGGCCAGACTCAGAGGACAATGTAT 2880

S D W L P K L D P V P A E A L K W R G Q T Q R T M Y

2881 CATCACTATGGTGAGCCATGGGAGTTAGATGGACATGGACTGATTGTTCTGACAAACTCCCAATATAATCCTAGAAATAA 2960

H H Y G E P W E L D G H G L I V L T N S Q Y N P R N N

2961 TAATTTCAAAAAGGAGTTAAAGAATCAAGCAGGCAGGGAATATATCGAAGAGTTAAAAGACAGAGTAAAAAAGTTAAAGG 3040

N F K K E L K N Q A G R E Y I E E L K D R V K K L K G

3041 GATCGGAGAAAGGAACGCGGGTCCTGATCACCAGTGGTGGAGAGTCTGGGCACTTTGCTATCATACATGTTCCAGTGCCG 3120

S E K G T R V L I T S G G E S G H F A I I H V P V P

3121 GGATATCCGAAAGGACAACCTTCAGAGGAGTATGAGCAACAGTTGGTTGATGCTCTAGAAGAAGGACTGCGTAGTGCCAG 3200

G Y P K G Q P S E E Y E Q Q L V D A L E E G L R S A S

3201 CAATGCAGGATATGCACGAGTGACAATGTGTGTTGATGGGATCAGATCAGGCGATTTGCCATGGATTGTGGCTGAACAGG 3280

N A G Y A R V T M C V D G I R S G D L P W I V A E Q A

3281 CCATGGTACGAGCTGGGCGTCTACACACCACTGGGGCAGCGGCCTGGTCTGGGATCACAGAATTGGTCCTGGTCACCTCC 3360

M V R A G R L H T T G A A A W S G I T E L V L V T S

3361 AAGAAGCAGCATAAAGAGTTCCGGGAGCGTCTTTCCGTGAAGCTGAGGCGGCAAGGAGGTGAGAATTCCTGGGTTCCGGT 3440

K K Q H K E F R E R L S V K L R R Q G G E N S W V P V

3441 GGCCAGAGAGACGCCGGGAGCCCCGCTGAGGACATCGCGGCTTAAAAGTACTTCAACCCCGGGCAAAGATGACGGGGAAG 3520

A R E T P G A P L R T S R L K S T S T P G K D D G E E

3521 AACCATCGACATCTCAACAACAGCCCCCCTCAGTTAAGACACCAGCGCGGATGAAGCCTGAGGCAGTAACCCCAAGGGTG 3600

P S T S Q Q Q P P S V K T P A R M K P E A V T P R V

3601 AAGGCCTCCCCTGCACGACTTAGCCTGCAACCAATGAACGATTACGTTGGTATGCGGACTGAGGGAAATACAGGGGTTGC 3680

K A S P A R L S L Q P M N D Y V G M R T E G N T G V A

3681 TACCTTCACCTACAGGGTGCCATCCCCCACCAACGTGCCAGAGGTGGTCAACACATCGATTCGTGGGCCAGCAGTCACAC 3760

T F T Y R V P S P T N V P E V V N T S I R G P A V T P

3761 CACGGCAGGAGGCACAGAGGGAGCCGATGATCTCGTCCAGCTATGCAGGACGACAGCCGGAATACAGTGACATCAGTGAA 3840

R Q E A Q R E P M I S S S Y A G R Q P E Y S D I S E

3841 GACAGTAGCAGTGATGGTGAAGGAGAGAAAGAAAGTGAGGAAGAGGTGGATGCCATGACTTCTGCACGACAGGAGGATAT 3920

D S S S D G E G E K E S E E E V D A M T S A R Q E D I

3921 CACCGGAGCGGTTGAGCTGAGGGTAGGACAGAGGAAAGGGACAAAGAAGAAGACCCCAGTGTACTCTGTCTATCGCACCT 4000

T G A V E L R V G Q R K G T K K K T P V Y S V Y R T S

4001 CAGAGAAGATGGCCCGGGATCTCTCCTTCGGGTGTGTGGAAACCAGAGATGGACGGAAGGCGTATATGCCCGAGGCCGGC 4080

E K M A R D L S F G C V E T R D G R K A Y M P E A G

4081 CAGCAGGATTATGGAATACCAATAGAATGGGCAGAACGACTCGAAGAAGAGGTCGTGTTGCCAGTGAAAGCACCAGTAAG 4160

Q Q D Y G I P I E W A E R L E E E V V L P V K A P V R

4161 GGAATGGGCTAACTTCCTAATGGCTCCTACCTACCCCACACTGAGAAGCCATGAGCCGCTAACAACTAATTTCAAGAATG 4240

E W A N F L M A P T Y P T L R S H E P L T T N F K N A

4241 CCTTTCTAGCCATCACACACGATGTCATGCTCCGTCGCTTGAAGAAATCTGCCCTCAAATATGCAAGGGAGACAGAAATT 4320

F L A I T H D V M L R R L K K S A L K Y A R E T E I

4321 CAACGCCAGCGAGAGCTATCCTCTAGTGATGAGGAAGAGCCAAGGATACCCCTCCAACCAAGAGCTGAGCTGCAGGCACG 4400

Q R Q R E L S S S D E E E P R I P L Q P R A E L Q A R

4401 CCGGACCACCAGAGATGTAGCGGTGGATGCTAACGGAGTTGGCGCGCCAGTAGTGACGGGAGGAGGTTCGAGCTTTGAGG 4480

R T T R D V A V D A N G V G A P V V T G G G S S F E E

4481 AATTCAAAAAGATAAAGGCGCTGGTGCAGGAGAGATCTCCGACTCAGAGTCTACAGGAGTACCTGAAGCAACACTGGCAG 4560

F K K I K A L V Q E R S P T Q S L Q E Y L K Q H W Q

4561 ATGGTAATGACCTGCACCCAAGATAACCAAGCACGACTAATGTGGCTAGCCCGTGCCACTGGGCAAGCATTAGGCCCAGA 4640

M V M T C T Q D N Q A R L M W L A R A T G Q A L G P E

4641 GGAATCAGCTGAAGAACATTACAAAAGACTGGTCTTGGAGGACTTCGACGACGAAGACACCAAGATAGAAAAGGCACGGG 4720

E S A E E H Y K R L V L E D F D D E D T K I E K A R G

4721 GACAACTAGACCAAGGACAGACCCTGTCACAAGTGTGGCATGTTTTGAGTAATGAAATCCCCGTAGGTCGGAATGTGCAG 4800

Q L D Q G Q T L S Q V W H V L S N E I P V G R N V Q

4801 GCGCTCAGGAGGCTGATGAAGGGCCGTAAGGGTGAAATGGATTTCGTCACCCTGCATCCTGCAAACAGCACACAGGAACA 4880

A L R R L M K G R K G E M D F V T L H P A N S T Q E Q

4881 GATAAGGGAGGTGGTGAAAAAGTGGGACCTCAAGAGGAAGTTTGAGGAACAAAGGCGGGCCGAAGAGGGTCAACGTACTC 4960

```
     I R E V V K K W D L K R K F E E Q R R A E E G Q R T Q
4961 AACAGAAGGCTGCCACCCCGAAACCCCCACCAAACCCACAGGGTCAGCAAAGGAAGACAGATCAACGACCTGCCTTCCAA 5040
     Q K A A T P K P P P N P Q G Q Q R K T D Q R P A F Q
5041 GGTCCACCCAAGAATGCCACGAGCAGCCGGCCATCTGACCAGAGACCACCAAAGGGGGATTCTACAAATAAGAGGCCTGG 5120
     G P P K N A T S S R P S D Q R P P K G D S T N K R P G
5121 ATATCTGCCTTTCGAGGAGTTTAGGAAATTAACGCCAGAACAGAAGGACGCCCTCCGACTCGCCCGCCAGCAGACGGCTG 5200
     Y L P F E E F R K L T P E Q K D A L R L A R Q Q T A A
       Gag ORF end          Pol ORF
5201 CGGGAGGGAAGAAGGAGTAATGGACACGACAGCTCAAGTCACCCTGGAGCATGCTTACCGGGTCAAGGATAAGATCTACA 5280
     G G K K E *
                     M D T T A Q V T L E H A Y R V K D K I Y T
5281 CCCAGGTCGATGGAGCTCCGTATTTGGTGGACTCAGGGGCTGAGGTGTCAATGACACGGAGGGCTCTGAGTCCTAAAGGG 5360
     Q V D G A P Y L V D S G A E V S M T R R A L S P K G
5361 AAGTTGACGGTGCAGTTGGCGGATGCCTCCATCGCTGAAGTCCCCTATGGCATCTGGAGGGGGATTATATGGATCCTGGG 5440
     K L T V Q L A D A S I A E V P Y G I W R G I I W I L G
5441 GACCTATGACTTGGTAACCATCACGGATTTAGAAGGATTACATCAGAGACCACGAAACCGCAGGAAGGTGGAGGAACGTC 5520
     T Y D L V T I T D L E G L H Q R P R N R R K V E E R L
5521 TGAAGATTTTGCAGGCGCGCCTAGTCAAGATACCTACGGTCGAGGACCAGGTCGGAGGGGCCTGGTACAAACCGGTTAAT 5600
     K I L Q A R L V K I P T V E D Q V G G A W Y K P V N
5601 GTTCCAGAGGTGACAGAGCAGAAATTACAGGAGAGTGATCTCTCTCCAGAAGGGAAACAGCGGTTGAGGGAGATCATCTC 5680
     V P E V T E Q K L Q E S D L S P E G K Q R L R E I I S
5681 CAAAGCAAAAGTAGCGAGATTTAAAAATGACTGTGGCGATCTAGGAACCAAATACATCCATACCATAGAAGGGGGAGTAC 5760
                      Aspartic protease
     K A K V A R F K N D C G D L G T K Y I H T I E G G V H
5761 ATCCACCAGTTCGACAGTACCCCCTGAATCCGGGAGCAGTGAAAGAAATGGACCTCATAGTCAGAGAATTACTGACTCTG 5840
     P P V R Q Y P L N P G A V K E M D L I V R E L L T L
5841 GGAGTAATTCGTCAGGAATTAAATCCAATCACTAACAGCCCGATACAGGCAGTAAAAAAGCCAGAATCCTCAGGAGGAGG 5920
     G V I R Q E L N P I T N S P I Q A V K K P E S S G G G
5921 CTGGCGACCTGTGATTAACTTCAAGGCTCTGAATAGAAGAACTGTAGCTAACAGAGCAAGCCTAATCAACCCGCAAGGGA 6000
     W R P V I N F K A L N R R T V A N R A S L I N P Q G T
6001 CTTTAAAGACCCTAAAGCTAAAGCCCTTTAAATCCTGTATAGACTTGGCAAATGGATTTTTCTCCCTACGACTGACACGA 6080
     L K T L K L K P F K S C I D L A N G F F S L R L T R
6081 GGATCCCAGGGGAAGACGGCCTTCACCCACAAGGGAAGATCCTACGTTTGGGAGAGGCTTCCACAAGGCTACAAAAATTC 6160
                 Reverse transcriptase domain
     G S Q G K T A F T H K G R S Y V W E R L P Q G Y K N S
6161 TCCCAACGTGTTCCAAGCAGCAGTTATGGATGTACTTGATGGATTAGGTGCCACTATTTATATAGATGATGTGTTCATTG 6240
```

P N V F Q A A V M D V L D G L G A T I Y I D D V F I A

6241 CAGATGACACGGAGGAGCAGCACTTGGACAGATTGCAGAAGATCGTGGAACGACTAACCGCTGCAGGACTCAAGCTGAAC 6320

D D T E E Q H L D R L Q K I V E R L T A A G L K L N

6321 TTGAAGAAGTGTCAGTTTGGGCGAAAGCGAGTGGACTATCTAGGATTCCAGGTGTCTGACGATTTGGGACTATCTGAAGT 6400

L K K C Q F G R K R V D Y L G F Q V S D D L G L S E V

6401 GTACAGAAAAAAGATCGAGCAGATAGAACCTCCGTCGTCACTGAATGAACTGCAAAAAGTGTTAGGACTATGCAACTATG 6480

Y R K K I E Q I E P P S S L N E L Q K V L G L C N Y V

6481 TGAGAGACTATGTTCCAGGATATCAGAGGTATGCTAAACCCCTGTATGCCAAACTAAAGAAACCAGATGAGACTGCAGCA 6560

R D Y V P G Y Q R Y A K P L Y A K L K K P D E T A A

6561 TGGAATTGGACAGCGGCTGATCAAAGCAACTTGGAAGAGCTGAGACAAGCGATCCAAGGGGCTCTGAGGCTTGAACCCCG 6640

W N W T A A D Q S N L E E L R Q A I Q G A L R L E P R

6641 AAGTCCAACGGCAAAGTTGGTTGCAGAAATAAGCTGCGATGAGGATAGTGCTACAGTAAAGGTGAGCAATGAGGGCGGTG 6720

S P T A K L V A E I S C D E D S A T V K V S N E G G G

6721 GACTAGTCACCTTATGGAGTTATGCTCTGACTTCGGTGGAAGTGAAGTTTCCCCCTGAAGAGAAGGAGCTTGCGGTGCTT 6800

L V T L W S Y A L T S V E V K F P P E E K E L A V L

6801 GCACGGTACTGGAGCTCCTTGAAGGATTTGGCGCAAGGCCAGGGAATCCGAGTCATCACCGAAAGCCAAGTACACCGTTT 6880

A R Y W S S L K D L A Q G Q G I R V I T E S Q V H R F

6881 CCTCAGAAAAGCCACTATTGAAAGCACTAAAGCAACTAACGCCAGATGGGGCCGGTGGGAAGACATCCTACTGGATCCAG 6960

L R K A T I E S T K A T N A R W G R W E D I L L D P D

6961 ATCTCGAGATTGGACCACTACAACCGGGAGTTCCAAAAGCTGCAACAGCTGAGAAACCGACGCAAGAGGAGTACGATTGG 7040

L E I G P L Q P G V P K A A T A E K P T Q E E Y D W

7041 ACTCTTTTCACAGACGGCTCGAGAAAAGGACCTGACGACACCGCGTACTGGGGATTTATTCTCAAGCTGAAAGGAAAGGA 7120

T L F T D G S R K G P D D T A Y W G F I L K L K G K E

7121 ACAGACCCGCCAGCGAGGGAAAGCACCCGGCAGCGCGCAGGCTGGGGAAGTGACAGCAGTGTTGGAGGGCCTGTTAGAGC 7200

Q T R Q R G K A P G S A Q A G E V T A V L E G L L E L

7201 TGGAAAAGAGGAAGATTAAACGAGCAAAAATAATAACAGACAGTTATTATTGCGCTCAAGCGTTAAAAGAAGATCTGACC 7280

RNaseH domain

E K R K I K R A K I I T D S Y Y C A Q A L K E D L T

7281 ATTTGGGAAGAAAATGGATATGAAACAGCGAAAGGAAAGAAAGTAGCCCATGAAGATTTGTGGAAAAAGATTGCCGAGCT 7360

I W E E N G Y E T A K G K K V A H E D L W K K I A E L

7361 ACGAATGAACCTGGATTTGGAGGTTGTACATCAGAAGGCCCATGTTAAAGAAGGAGCTCACTGGCGAGGAAACGAAGAAG 7440

R M N L D L E V V H Q K A H V K E G A H W R G N E E V

7441 TGGATCGTTTCGTCCAGATGAGAAAAATTGTCTTTGTCGGGATCGAGAAATGGGACAACACGCCTCAGGGTAAGCTTGTC 7520

D R F V Q M R K I V F V G I E K W D N T P Q G K L V

7521 CCGAAAGACTGCGTGAAGGGGGTAACGGAAGCAGTGCATGAGGCACTAGGCCATGCCGGAGCTGACCCGACGCGCAGGGA 7600

```
      P  K  D  C  V  K  G  V  T  E  A  V  H  E  A  L  G  H  A  G  A  D  P  T  R  R  E
7601 GCTGAAGAAACAGCAGCTATGGATTCCAGTACGAGAAATCCGGCGTGTTCTCAAAGACTGTGTTGAGTGTGGTCGGTACA 7680
       L  K  K  Q  Q  L  W  I  P  V  R  E  I  R  R  V  L  K  D  C  V  E  C  G  R  Y  N
7681 ACGCAGGTCGGCGGGGGCAGCGCATGGGAGGACTCACCATCAAGAGCACTGTTCCCTGGGGGTCGATTTGTATGGATGTA 7760
       A  G  R  R  G  Q  R  M  G  G  L  T  I  K  S  T  V  P  W  G  S  I  C  M  D  V
7761 GCAGGACCCTTAGGGATAACAGGTATGAAAGGAGAAAAATATCTGTTAGTGTTGGTGGACTCTATGTCAGGGTATGTTGC 7840
     A  G  P  L  G  I  T  G  M  K  G  E  K  Y  L  L  V  L  V  D  S  M  S  G  Y  V  A
7841 TGTGAAACCTGTGCGCCAAGCAAATGGTTCCAGTGTAGTCAGCATGCTGGATCAGGCATGCTGCTATATGGGAATACCCA 7920
                                 Integrase core domain
       V  K  P  V  R  Q  A  N  G  S  S  V  V  S  M  L  D  Q  A  C  C  Y  M  G  I  P  K
7921 AGGAGCTAAGAACGGACAATGGAACGCACTTTCGGAACGCTAAGGTGGACCAGTGGTGTCAAGAGAATGGGGTGATGAGA 8000
       E  L  R  T  D  N  G  T  H  F  R  N  A  K  V  D  Q  W  C  Q  E  N  G  V  M  R
8001 GTGTATTCACCACCATATACACCGCAGGCCAACGGAGTGGTCGAGCGAACCATTGGACTGGTAAAGAGCTGGATTGGTAA 8080
     V  Y  S  P  P  Y  T  P  Q  A  N  G  V  V  E  R  T  I  G  L  V  K  S  W  I  G  K
8081 AAATGCTAATGGTAAAGAATGGAGTACAAAAGTCCTAGAGCTAGTCAAGACCCTGAATGACAGGCATCGACAAGGGAGGC 8160
       N  A  N  G  K  E  W  S  T  K  V  L  E  L  V  K  T  L  N  D  R  H  R  Q  G  R  P
8161 CCTCGCCAGCAGAGGAATTGAATCAACGCCCCTTCACGTCACAAGAGGCGGGCAGGAGCTCAGATGACAAGAGGCCGTCC 8240
       S  P  A  E  E  L  N  Q  R  P  F  T  S  Q  E  A  G  R  S  S  D  D  K  R  P  S
8241 CCCGATGACAAAATGTCACTGCAGGTTGGACAGCGAGTATGGATTAAAGCACAGGCTCACCCAGCTGGAGCAGCTGTGAA 8320
     P  D  D  K  M  S  L  Q  V  G  Q  R  V  W  I  K  A  Q  A  H  P  A  G  A  A  V  K
8321 AGCAAAGTACGACAAGACTGACATAGTGACTGAGATCTTGGACCGAAACACGGTTCGGTTGAAGAAGTCAGGAATCCAAG 8400
       A  K  Y  D  K  T  D  I  V  T  E  I  L  D  R  N  T  V  R  L  K  K  S  G  I  Q  G
                Pol ORF end                                Acc3 ORF
8401 GAGTAGAGCAGCTGAAACCAGTTCCTGATTAGGTGACCTAGGAACCAAAAACCATGGCCAGTAGCACACCTTGCCTACCA 8480
       V  E  Q  L  K  P  V  P  D  *
                                                           M  A  S  S  T  P  C  L  P
8481 CCCTATGTCAGACTGGCCACTGTTAAGGGGGTGGTTATCATGAGACGCAGTACCGATGGGCTCTGGGCAGGCAGAGCCCT 8560
     P  Y  V  R  L  A  T  V  K  G  V  V  I  M  R  R  S  T  D  G  L  W  A  G  R  A  L
8561 TGAAATGTTGGAGTTTGCCAAAGAAGGAATCTTTACAAGTGACAAAGAGTTCCTGCAATGGAAAGAAATTAGAAATAACT 8640
       E  M  L  E  F  A  K  E  G  I  F  T  S  D  K  E  F  L  Q  W  K  E  I  R  N  N  C
8641 GTGTGGTGTGTTGGAAACCCACCACCCTGGGTGTCCAGTGGGAAGGCCCAGGAAGGAATAAGCCCCTCCGGGCTGAAGCA 8720
       V  V  C  W  K  P  T  T  L  G  V  Q  W  E  G  P  G  R  N  K  P  L  R  A  E  A
8721 AATCGGAGGGAATTCAGGGGGATGCACCCCAAACCAGTAAAAGTGCTGGATGGTCATGTGTGTGGACTATGCCGGCTGAA 8800
     N  R  R  E  F  R  G  M  H  P  K  P  V  K  V  L  D  G  H  V  C  G  L  C  R  L  N
8801 TGGTTTACCCCGACTGAAGTTTTACACTCAGTGGGATTTAAGAGGAGGAAAAGTGGACTGCAGAGAGTGTACCTGCCAGT 8880
```

G L P R L K F Y T Q W D L R G G K V D C R E C T C Q W

8881 GGGATGGTGAACGACTCCACCCATGTGGCAACTGCAAGCTGCAGCAAGAGAGTGGACGTCTGCTGCAGGTCGCCACCGCA 8960

D G E R L H P C G N C K L Q Q E S G R L L Q V A T A

8961 GAGGGATGGCAGGTGAGGAAATTCCTGGACCCTCTGCAGCGCTTCACAACAGCGACGGTGGAAGGAGGAGAACGAGGGGA 9040

E G W Q V R K F L D P L Q R F T T A T V E G G E R G E

9041 GAGCGGGAGTGACCCACTCAGTTCCCCTGATCCCGGCTCGCCAGAGACCGAGGGCCCTTCTGCACCACCCCTGCCACCGG 9120

S G S D P L S S P D P G S P E T E G P S A P P L P P E

9121 AACCCTCATCAGCGAGAGGGCGAGGGAAAGGAGCAGCGAGACATCCTGAGCAGGAGCAGCTGTGGAAGGAGGCGATTAAG 9200

P S S A R G R G K G A A R H P E Q E Q L W K E A I K

9201 AGAGGATGGCCTTACAGTCCGGCTCGGACGGAGATCCACGAGCCACCGCCATCAATGGAGCAGGTGAATATGGTGTTTCG 9280

R G W P Y S P A R T E I H E P P P S M E Q V N M V F R

9281 AGTGCAAAGGGACCCCGATGACCCAGGATACTCCCCTATCTCGAGAGATCTGAGAGAAGATCAGCGGGAGATGGCGGAGA 9360

V Q R D P D D P G Y S P I S R D L R E D Q R E M A E S

9361 GCCAGGGCTGGACTGGACCATGGGAGCTGACCACTTGGGGCCGTCTAATAGAGGAGGTTTACTCTGATCCTTCCCTACCG 9440

Q G W T G P W E L T T W G R L I E E V Y S D P S L P

9441 GTCCAAGATTTGGAACCAGTCCCAAGAGAGGCCGGGGCGAGAAGCACCAACCTGTTCTACAGTACCACAAGCCTGGATGC 9520

V Q D L E P V P R E A G A R S T N L F Y S T T S L D A

9521 ATGGGGCCACCAGGCGGCGCGAGCCAAAGAAAAGAGTAAGGAGTATGTGGTGACGCCAGAGGCGTGGCGAAGAGAAGGGG 9600

W G H Q A A R A K E K S K E Y V V T P E A W R R E G V

9601 TGGTCCAACTGCCTGGGACGTGGGCAGGGCGTGCGGCCACGCCCACCTACCCTGAGCAGCTGACCAGGGTACGAGTGGCG 9680

V Q L P G T W A G R A A T P T Y P E Q L T R V R V A

9681 GTTCAGACTGTGATCATTCCCTTCCGAGGAGTCTACTCAGTCGGAGCCACAGTGACGTGCCTGGGAGAACCACCAAAGCC 9760

V Q T V I I P F R G V Y S V G A T V T C L G E P P K P

Acc4 ORF

9761 TGAAGAGGCAGAAAAAGAGGGGAAAGGGAAGTGGAACCCTTTCCAACGATTCAAGGTGAAGAAATGGAGCCGCAAGTCCC 9840

E E A E K E G K G K W N P F Q R F K V K K W S R K S Q

M E P Q V P

Acc3 ORF end

9841 AGCAGTAGCCGATACTGGGACACAGGGACGGGCACGGAGGCGGAAAGAAAGGTGGCAGAGGAAGCTGGCCCGTGTACCCT 9920

Q *

A V A D T G T Q G R A R R R K E R W Q R K L A R V P Y

9921 ATATAAGGCTGCCAAATCAGAAGGTATGGTTAAAATACCCGAAGAGTGACTATTCATATAATTGGATGTATTGTGGATCT 10000

I R L P N Q K V W L K Y P K S D Y S Y N W M Y C G S

10001 GCAGCGTATTGCTGTAGTACCGGGGAAGTAACCAGATCAGCCGACTTATCAAAGCCTGAAGAAGGGCAAATGGGAGGTAA 10080

A A Y C C S T G E V T R S A D L S K P E E G Q M G G K

10081 AGTGTTTCGTGGACATGAGTTGCAGTTCTTCTCGTTGAACTATGAACTTTTCAAGGGCCGATGGAAGAACCAGCCCGTGG 10160

V F R G H E L Q F F S L N Y E L F K G R W K N Q P V V

10161 TGGGGTGCCCCGAGTCGATGCCCTGGGTCCTTAATACAGTGTACCTTCAAGTCAACCACGAGCTGTTCAAGCTCCGCTGG 10240

G C P E S M P W V L N T V Y L Q V N H E L F K L R W

10241 GGGGTTAATCACTGCCAACCTTGGTTAGGGACGTATGTGGAAATATGGCTCGGAAGCCTGCTTATATGGACTAACAGCCC 10320

G V N H C Q P W L G T Y V E I W L G S L L I W T N S P

10321 TCACACTCACCAGAAGTTGCAGCAGAGAATTAAAGATCCAGAGCGGGTTGTAATGGAAAATCAGCACCTGCCTCTGAAAG 10400

H T H Q K L Q Q R I K D P E R V V M E N Q H L P L K V

10401 TGCAGGAGAAGAACTTTCCCAATTCTCGCCAGTCTATGATAAGTCTACCCAAGACACGGAGGGATGAGCCTCACCCTCTT 10480

Q E K N F P N S R Q S M I S L P K T R R D E P H P L

10481 CTGCCAAGCCCTGACAGATGGGGAAAATTGGTGGAATTGCTGGACCGCCGAGGTGAGCCCTTAGTTGCTGAGCTGGCAAT 10560

L P S P D R W G K L V E L L D R R G E P L V A E L A M

10561 GGTACGGGCTGAGGAACCTCGTCGGCCGACGGGAAGAGTTATCATTACCCGCTACACTTGGATGTGGAAGGGCTTCGACC 10640

V R A E E P R R P T G R V I I T R Y T W M W K G F D P

10641 CATGTATCAGGGACCATTACAAGATTCACAATTGTGACAAAGATAAGCATCCTTATCCGTTGATGAGACCACTCATGATA 10720

C I R D H Y K I H N C D K D K H P Y P L M R P L M I

10721 GCACGGCTGTGGCGGGATCCATCAGGACATCTGCACTCACCACGACCCAAGGTAACAGTGGTTCCTCTGTCACCTGGAGA 10800

A R L W R D P S G H L H S P R P K V T V V P L S P G D

Acc4 ORF end

10801 CAGGGGGAGGAGCTACTGGCTGTGATTAACACTGAGCCCCGTAAGGCCTAAATCCATTCACCACCTGAGGCCTTGAGAAG 10880

R G R S Y W L *

Env ORF

10881 TGAGCAGCATGGCTGGAAGAACTCTATTCTGTCCTGCAAAATGGATTATTTTGAAAACTATTGTCGGCTTTCTGGGACTT 10960

M A G R T L F C P A K W I I L K T I V G F L G L

10961 TCTGCTATTTTGATATTTTTGTATTTTCATGGAAATGGACTCTGGGAGAGTGATCATGTAAGGATCAGCTACACTTCCAC 11040

S A I L I F L Y F H G N G L W E S D H V R I S Y T S T

11041 TGAAAGGTATAACCAAACCTGTTGGAATGTCAAAAAGGAAATAGTCACAATAAGGAATTTTTCCCTGTTTTGTGATGAAG 11120

E R Y N Q T C W N V K K E I V T I R N F S L F C D E D

11121 ATGAAGAGGACAACTGCACGGAAGCAGTGTCTGCTTTTAATGTTTCAGTGTTACTGCAATTCTTTGACTCCCATAGAGGC 11200

E E D N C T E A V S A F N V S V L L Q F F D S H R G

11201 GAAGGAGGGGGAGTAATCGAGGCGATTGACTTTCAAGGTGAGACACTGCTTTTGCATGGCATTATCCCCCAACAAAATGA 11280

E G G G V I E A I D F Q G E T L L L H G I I P Q Q N E

11281 GACCCGTATGGTGCAAGCTAATTGGATTCAAGTGGACGGACAGAACCCGTATTGGTACTACACGCAGGCATGTGTCAAAG 11360

T R M V Q A N W I Q V D G Q N P Y W Y Y T Q A C V K G

11361 GAAGGAAGATAACCTACTGGGCTGAAGAGGTAACTCTGCCAGCTCACAATTTTCAGCAACAATGTCATGCAACGATCCCG 11440

R K I T Y W A E E V T L P A H N F Q Q Q C H A T I P

11441 AAGCCGGAGGTGTTTTGGGGAAATCTGAATCTGACATGTGATATGCAGACTCAACTTGAAGGGCATGAAGATAATGGCAC 11520

K P E V F W G N L N L T C D M Q T Q L E G H E D N G T

11521 CGGGAAGTTCCCCGTAGCCCACGGGAATAACTCTACAACAGCCAGCAACTCCACACCGAAGCCTGGAACGAGGAAAAGGC 11600

G K F P V A H G N N S T T A S N S T P K P G T R K R P

11601 CACAACCGGTCCAGAAAAGGCCTCCCCAGGTTTCAGAAGCTGACCAGAAAAGAATGAGGAAATGCATGGAGGAGAAGGTC 11680

Q P V Q K R P P Q V S E A D Q K R M R K C M E E K V

11681 TTCAAGCTTAATCCTTGTTACTGGAGTTTGGGGCCTATAGTGTGCAAAAGACAGCACTATCTGGTTCTGAGCCAGGCCGA 11760

F K L N P C Y W S L G P I V C K R Q H Y L V L S Q A E

11761 GGAAAAGACTTGGAGAGTCTGGAGCAATGACTGGTTCGGGAGAGGGACAAGAAGGAAGGGAGATCCACCCGATTCTGGGG 11840

E K T W R V W S N D W F G R G T R R K G D P P D S G G

11841 GGATCTGGATTGACGACTGGCTGAATCAGAGTCTACCGTGGGTGTGGAAGAGAGGAGTAACCCATCTGAGGGCAACTGCT 11920

I W I D D W L N Q S L P W V W K R G V T H L R A T A

11921 GACACTGTGACGCAGCCCAACCAACTGGGAGGCCCAGAGAGGTCGGAAATGTATGTCCAAAGAATCCCAACACCTGATGA 12000

D T V T Q P N Q L G G P E R S E M Y V Q R I P T P D E

12001 GTTATGGTCTGCACCATCAGGAGACTTTGATAAAATTGACCGATGTGTCCTCCAGAAAATGGCGGCAGATCTTGACGGGA 12080

L W S A P S G D F D K I D R C V L Q K M A A D L D G K

12081 AAGACTACTCGAAAGGTGAAGGAACAGTGTCACAGTATTGGGCGTCGCCAAGGTGTGACATTACCCAAGTGGGAGCATGG 12160

D Y S K G E G T V S Q Y W A S P R C D I T Q V G A W

12161 AGTGAGGGATATTGGAAGTACAATTGTTCCCGAATGAAAAGCCATAACACCATGAAGGGGGTGCTGGAGCAGTGGCCGCT 12240

S E G Y W K Y N C S R M K S H N T M K G V L E Q W P L

12241 GTTTTGGGATGCCCCTGATAAGACTCGAACATGGTGGGGTCTGCAGAAAGAGGAAACCGAAGCCTGGGTTTTGCCGTGCC 12320

F W D A P D K T R T W W G L Q K E E T E A W V L P C L

12321 TGTCACAAACGAAGAATTATTGGATTAAGTATCAATACGTGGCTACAGTCTATTCCACAGGGGAAGGACTCTATGAATGG 12400

S Q T K N Y W I K Y Q Y V A T V Y S T G E G L Y E W

12401 TATAGATGGACTGGCATCAGACCAAGTGTGACCTTAACCCCGAGACCGTGGAAAATGAGATGTAATCGAGCCAACGACAC 12480

Y R W T G I R P S V T L T P R P W K M R C N R A N D T

12481 CTGCACCATAGAGCTGGCACGTAGACCCTGCAGTGAGTTAGATGGCTGGAGTAATGACTGTACTGAGTGGTATGGCGGAG 12560

C T I E L A R R P C S E L D G W S N D C T E W Y G G A

12561 CTTACGAGGCCTATCAGCAGGGCGCCACCTGGGTAAGAAACGATGTCCATTTGTTTGGAAATCTGACCAGAAGGAAGCAG 12640

Y E A Y Q Q G A T W V R N D V H L F G N L T R R K Q

12641 AAACAACCAGAGTGGCAACGGGCACCAGGACCAGGGAGGTACGGTTGTGTAAAAGCCCTACTAGGTCCAATGGTAAATCA 12720

K Q P E W Q R A P G P G R Y G C V K A L L G P M V N Q

12721 GCGCCAACTGACTGGGAAAATAGTGCCTGCAGTAAGTAAAGCAGAACTCACCAGGCTGGAGGAATATGCATTTCGCAAAA 12800

R Q L T G K I V P A V S K A E L T R L E E Y A F R K S

```
12801 GCCTGTGCGATGGCAAAGAAGTAGAGGGGTTCGAATGGATCGGACCTAATAAAATGTACAACAATGGAACTTGTGAAATG 12880
       L  C  D  G  K  E  V  E  G  F  E  W  I  G  P  N  K  M  Y  N  N  G  T  C  E  M
12881 CCACAGGAACATTGGTTGCAGTGTAATGGGCGCGACTGTACAGTGGCAGAGCTGTACGGAAGAACCGTGCTCGAGGGTAT 12960
      P  Q  E  H  W  L  Q  C  N  G  R  D  C  T  V  A  E  L  Y  G  R  T  V  L  E  G  I
12961 CGAGGAAGTACCCAAGGAAATAGTGGAGAAGATTGAAGATGGAGCAAAAGCTCTGGGATCAGAGCTTTGGGCGATTATGA 13040
       E  E  V  P  K  E  I  V  E  K  I  E  D  G  A  K  A  L  G  S  E  L  W  A  I  M  K
13041 AATCACTTCTGCCCTACGCCTTGATTGGAATAGGAGTCGTTCTGTTGGTGCCACTCCTAGGAGGTCTGATCAAGTCGGGA 13120
       S  L  L  P  Y  A  L  I  G  I  G  V  V  L  L  V  P  L  L  G  G  L  I  K  S  G
13121 GTCAGGGGACTCTGCAGAAGGGGGCGGAGGAAAGGAGCTACGCCCAGAAACCGGGAGGAGTTGGAGCTCCTCCTACGCGA 13200
      V  R  G  L  C  R  R  G  R  R  K  G  A  T  P  R  N  R  E  E  L  E  L  L  L  R  E
         Env ORF end                                    Acc1a ORF
13201 GGAAGGATCACCTCGCGAGTAAAGGGTTCTAATTGCCAACCTGAACCCACAATGGGACCTGCCTGCGTGACGAGGGAGAC 13280
       E  G  S  P  R  E  *
                                                    M  G  P  A  C  V  T  R  E  T
13281 CACGATTAGCATTGAAGCCTTCGACATGACCACAGAAACAGGAAATTGGACTGGATCAACCACAGCACCTCCCTGGCAGA 13360
       T  I  S  I  E  A  F  D  M  T  T  E  T  G  N  W  T  G  S  T  T  A  P  P  W  Q  I
13361 TTAAGGAGTTTAAGGGAAAAGTCACGGAGTTCGTCACCTTGGAAGTAATATGGTTTGTCTTCTGCTGCACGGGGCTCTGG 13440
       K  E  F  K  G  K  V  T  E  F  V  T  L  E  V  I  W  F  V  F  C  C  T  G  L  W
13441 GCTGGAAGTCAGGCATTCAGGGTCCTCTGCCTTGTTGAAGGAATGGTACGAGTACTGATAGTAAACGTATGGAGAGGTGA 13520
      A  G  S  Q  A  F  R  V  L  C  L  V  E  G  M  V  R  V  L  I  V  N  V  W  R  G  E
13521 GCCTACATACACCACGTCCTGGTTCGTTGCCTTCCTTCTCGGAAGGCTCAAGATCGACACAACCAAGAGAGGAATTATGA 13600
       P  T  Y  T  T  S  W  F  V  A  F  L  L  G  R  L  K  I  D  T  T  K  R  G  I  M  R
13601 GAATGATCATTCTAGTCATCTGGCTGGTACTGGACTTGGCCTCAATGTGGTACCCAGTAGCATATTGGGCTAGGCTGGTA 13680
       M  I  I  L  V  I  W  L  V  L  D  L  A  S  M  W  Y  P  V  A  Y  W  A  R  L  V
13681 CTAGAAGGACTGGTGGTTGTGGGCTTACTAAGCTACATGTACTCACAAGTCAGCAAGTTGAAAGGCAGCCATGCTTCAAG 13760
      L  E  G  L  V  V  V  G  L  L  S  Y  M  Y  S  Q  V  S  K  L  K  G  S  H  A  S  R
13761 GTGGGCACTCAAGTTACTTCGACTGTGCCTGTGGGGATTCGGTTGGGGAATTCTGAGTGTCATGAGTATACTGGAAGTCA 13840
       W  A  L  K  L  L  R  L  C  L  W  G  F  G  W  G  I  L  S  V  M  S  I  L  E  V  T
13841 CCACCAACCTACCCCTGATCGTCTGGTTCGTGCTGTATGGACATCTGTTTCCTCAACCAGTTCGGAAGGAGCCGGGTAGC 13920
       T  N  L  P  L  I  V  W  F  V  L  Y  G  H  L  F  P  Q  P  V  R  K  E  P  G  S
13921 GGTTCGCCCAGGGAGGAAAGAGCCAGGACAGCCGACACCCGACGTTGGACGAGGGGCGTGGAACACATGTATGAAGAAGT 14000
      G  S  P  R  E  E  R  A  R  T  A  D  T  R  R  W  T  R  G  V  E  H  M  Y  E  E  V
Acc1a ORF end
14001 CCCACTCTGAAAACGAACTGGAAGGCGTTAACCTCGTCACAAAGGCAGCCCAATGCATTTCGCAAGCTACAGCTATGTTA 14080
       P  L  *
```

14081 ATGATAATCATCCAATCAAAATTTATGTTACCATGTATGGATAAGAAAGTAAGCAAGAAGTGAAGGGAACCGCCCGTAGA 14160

14161 AGGCGGGGCTAAAGGATATAACCAGCGGAGCATAAGGAGGAAGTGGTCAGTTCGAGATCAGGAAGAGCAGAGGCAACACC 14240

Acc1b ORF

14241 ACAACGAGATGTCGAGCACCACCAGCAACGCAAGTACAATGTCTACAGCAACCACCGCCACCACTATGTTCATAGGAGTA 14320

M S S T T S N A S T M S T A T T A T T M F I G V

14321 AGTACAATTCCAACTTATCTCCAGAATTGCACTGAATCCCAAGAACCAGAGATCAAATTTACTAGAATTGATTTCTGGGT 14400

S T I P T Y L Q N C T E S Q E P E I K F T R I D F W V

14401 TTTCGAGTGTATATGGTTGGCATTCTGCTGCAGCGGAGATTGGTCTACAAAGCAACCCCTGAGGATTATAGCCCTGTTTA 14480

F E C I W L A F C C S G D W S T K Q P L R I I A L F I

14481 TCGGCTTCTTCAAAGCGTTGATGGAAATCATCATGCTGCAAAGAATGAAGATCACCCTGTCCTGGTTTGTCATCTACCTA 14560

G F F K A L M E I I M L Q R M K I T L S W F V I Y L

14561 CTGGGACGCACCGACATTGAAACCAACAAGAGAGGGGTAATCAAAACGGTAGCATTGCTGATTTGGTTGCTGCTCGACAT 14640

L G R T D I E T N K R G V I K T V A L L I W L L L D I

14641 CGCAGTGGACGTCATTGGAGACAACTGGAACATCGGATATCTGTTTGAAGGAGTATCACTGGCTGTCTTCTTGGCGTATC 14720

A V D V I G D N W N I G Y L F E G V S L A V F L A Y L

14721 TACTGACCTGGACGTTGGTCAGATATGGGAGGCCAGGATGGTGTCAGCTTGACCGGGTCATCATTGGAACCATATGGGCC 14800

L T W T L V R Y G R P G W C Q L D R V I I G T I W A

14801 CTGGGCTGGATAGTCCTGACGTCCGCCTGCTGTGCTGGGGCTGAGGCATCGGTCTCCATGGCGGGATGGTTCATGCTGTA 14880

L G W I V L T S A C C A G A E A S V S M A G W F M L Y

14881 TCCCATCTTCTACCCCCGAGTCCAAGCCAGAGTGGCCGGAAGAACCCCACCTCCACAAAGGAGGAGCGGCTGGCTTGACC 14960

P I F Y P R V Q A R V A G R T P P P Q R R S G W L D L

Acc1b ORF end

14961 TGATCGGCAGCAGGGTTCAGGGAGGAGGAACCCTAGCCTGATCCAAAAGAAGACTGACCAACGCTCTGCTTGCAAGAAAA 15040

I G S R V Q G G G T L A *

15041 TGAGAAAAAGAGTTGAAAAATGATTAATCATTATTAATTGATTTTGTTGTTTGATTATAGGAGGACGCAGGATGCAGCGG 15120

Acc2 ORF

15121 GTGCGGGGCTACGCTGACAGAGGCCGGCTGTTTGTGGAACCTGGGAATAAGAATGCAATGTTCCAGGTGCTACATGGAGG 15200

M Q C S R C Y M E D

15201 ATCCTCACCCGCATGCAGTCCATGGCGTCCTCCAAGGAACAATGACCACACAAGTACCAGTAGAAACGGCTATGGAGCTT 15280

P H P H A V H G V L Q G T M T T Q V P V E T A M E L

15281 GAGGAGCAAGCTAGAATCATCTGGAGTTCGTTCTGGAACAGAGGAACGACCTGGATACCACGCGTTGTCGTGGGAGGATT 15360

E E Q A R I I W S S F W N R G T T W I P R V V V G G F

15361 CTACATACCGACCTACACCCGAATTTGGGAGTTCGGTGACCGATCCCGCATTCGCACCACCCATTCTTTGGACGGGCGAA 15440

Y I P T Y T R I W E F G D R S R I R T T H S L D G R I

15441 TCCTGATAGCTGGAGCTCGGCAGCTGCCTACACCATACCAGGCAGAGCCTCGCACACCCCTGTCATCAGTACCACGGTCT 15520

L I A G A R Q L P T P Y Q A E P R T P L S S V P R S

15521 TCAGCGGAAGCTGGGAAGGAACATCGGGACGTAGAGACGCAAACGGACCTCGATGAAGCTGGTGTGGAGGACGGAGACGA 15600

S A E A G K E H R D V E T Q T D L D E A G V E D G D E

15601 GGGCAATGAGGCACACCTGGAAGAGATGGCTGAAGCTGTGGAACAACAGCTACAGGCGCCCCCTTCCACATCGGAGCCCT 15680

G N E A H L E E M A E A V E Q Q L Q A P P S T S E P L

15681 TGGATGTTCCGATAGAGGAGGCACCAACATTGGCAGCCATGCTGGAGCAGCTCGACGCGGCAGAATGGCCACGAGGGGCC 15760

D V P I E E A P T L A A M L E Q L D A A E W P R G A

15761 GCTCTAGGGCAGCAATCGGAGAATCAGCTCGAAAACATGGAAACGCCCAGAGAGGAATATGTTCCTGATGTTTTGGAAAT 15840

A L G Q Q S E N Q L E N M E T P R E E Y V P D V L E I

Acc2 ORF end **3′-LTR**

15841 AATTTCTGATGACTCTTGGGATGGTGATCTGTAAAATCATCGAAAGGTAACCTGCAATCTCAAGAAGGGGG**TG**TCGAATG 15920

I S D D S W D G D L *

15921 AAGTGGGTTGAGGTTGAGATCTTGGTTATTCGATGGCCTGTGCCTGGTACTATGTGGACAATCATAATTCTGATTTATGT 16000

16001 AAAATGATAAAGTTGATTAATGGAATGTAACAAATAATCTGTATGATTTCTAATCAAAGTTGAGAAGAGTGTGTGTAACA 16080

16081 CAGAAATGATTTGAGCACAGCATGTAAAGGAAGTTGTGAAATCTTGGAGAATCACTGAGCAGGGTAAAATGAGGAACTGA 16160

16161 AACCTGTTGCCTAGGAATCAGAAGGAACAGAAGGGAAAACAAGTTAAGAATAGTCTTAACTGGAAAAGTACCAAACAAGC 16240

16241 CATGTATTACAAGAAATGAAGTAATGGTCAAAGCCAAATACCATAATTGGTAATCAAACAGTGGAGTGATAATTTGTCAT 16320

16321 GATGATGTTTTAAAACTAGGATGTGTCTTAGACTTAAACTATAAAAGGCGATGCACAGAAGCATCGGGTGAGATTCTAGG 16400

16401 GAGCCCTTCCCAGCACCAGGACGAGTCTACAGATTGGACCAAGCCAACCCAGATGTGGACATTTAATTGACTTTAAACGT 16480

16481 CTTTGTGAGAATTCTCCCTGCATTGTCAGATGAGGATTGGAAGTACCTAAGCTGTTGGATTTCAAAGAGGATTTCCGAAG 16560

16561 AACAGTAAGGATTATAAAAGAAGGACAGTCAACTGAAGGGAGTCAACATCTCTCTTTTTGGACTTCTCCTTTTTCCCCCC 16640

16641 AACTTCACTGAGGCAGTAACGGACTGGACTTTGGGTGTGGTCGCTTCGGCCGAGTGACTCACTTGAAATTCAGTCTCTTC 16720

16721 AAAAAGGATTTATGGACAGAATTGGACATTTTCAAGCCCCTTCTTCATCAACCCAGCTCATAAGTAACCATTTTGATTGA 16800

16801 GATTAAGTTTTTGATTTATTCTGTTTTTGAAGTTTAGATTTTGAGCTGATTGTTGACATTCCAGAATTGATTGAGAATTG 16880

16881 ATTTTGAGCTAGATGCAAACTGCTTGAGCTGAAAACCTGCATAATAAAATTTGACTCTAAAAAGTCAGTATAATTCGTTG 16960

16961 AGAATTAATTCCCTTTTTAACGGTACACTTATGTCCATGCAACAGTAAAACTCTAGGCAAGTTAAGCTATAACATCTGGT 17040

17041 TCTATTTTGGACACACTAGTCGTAAGTAATCCACTTCAACTTAACTTATGTCCCTTGGGCTCACTGTCCGAGCCGATAAA 17120

17121 AGAAATCTGGTCGGTAACAAGCGCACTGATCAAAGGGGCTGGCTGCTATAACTATCTGGGCTGTATTTTGGGGTCGGCTG 17200

17201 CGTGAGCCTTCCTGACCGGCAGCATCAGAGTACAGAGGAAGCGGGGCAGATGTTAGGATTATTAGCAGTTAGAGCCAGAC 17280

17281 GAGCAGCCCCGCCACAGCTTAAGTAGTGTCACTGAGCTCAGAAGTGGGTAATACCCTGCTGGCCAAGTGATCAGGACCCT 17360

17361 ATGAAACGGAGTAGCTGTCACAGAGGTAAAATACCTGTGGGATCTAA**CA**

AliFLERV

5′-LTR

1 **TG**TCAAGAGACCCGACAGCTCCGGTCATCACTAGAATTGGAACCAGACCTATGGAGCCTTCACTTGACCCCCAGCAGAGG 80

81 AGGGAGTGTGACAGGAAAAGAAGCCACAGGAAGAATCGAGGTGAAGGCCGAGAAGAGAAGAAAATGAGGCGACAGGGAGG 160

161 AGGTGAAATGATTTGAAGTTAATCAATTTTAATGCTGGCAAAAATACATATGTAACTTGAGTAATGATTATGCTTAATTG 240

241 CAATGTGAATGATTAACTAATGTACTATGATGATTAATGTGTTTTCAAATGTAAAATGGTGATATAGAATAGAAAGAATC 320

321 AAAGTGTGTCCTAAGATGAAGGAAGGTTGTGTGTGTGTGCTGTTTTGGACGGTGAAAAGTACAGGGCACATTTCTGGATA 400

401 ATAGGAAAAACAGATGTGTGGGCTTTTCTGGAATGTCCCAGAGGCCCTGTGGTGAAAAACAAAGTGGTTTTAACTCACCA 480

481 CAGAAAAAGTCCCTAGCAGTCCCATAGCAGAAAGTCAGCAGGATTACCTCATTCTGGAAAGTACAGAAAAACACATGATG 560

561 GGAAGTCCCCACCCTTCCACAGAAAGGAAGGAGGGGGGCGAACGAGCCCAGAAAGAGTATAAAAGGCCAAGCAGTCCAGC 640

641 AGTAAAGCGTCTTCTGGCCATCGACTGGAGAAGAAGCAGCAAGCAGCGAGGACATCGAGAGCAGAGGAGCAAGCAGATCC 720

721 GAAGATCATCTCATCTGGGGCAAGCTGACAGATTCCCCTCCGGCGATCTAAGGACCGAAGACCAAATTGGACCCAGCCCA 800

801 ACTCTGAGAAGTTCTTTTCAGGCAACAATCTGATGCACCTCATGGGGGTAAAACCGACTGCTCTAACCTGTCCTGCCGTC 880

881 TCCAAATTAACAGAAAATGCCACACCACCTAATTAGAAAGATAGGTCAGCATATTAGAGCATTTTATTTTTATCACCATC 960

961 AATTATTAATCAATATTCTTAATCTGTATAATCAAATGTGGCTTTTCTTGCAAATTCCTATTAAACTGCTTACAGCATCA 1040

1041 TAAAGACAAATTCCTGACTGGACATTGATTTTTAAAGTTGTCCTTTAAAGTTTTTCTCTGATTACATGAGAGTTTTGTTA 1120

1121 CTGTAAAGCAACAAGGTAATAACATATTAAAGAGTATGGTTGCTTTGAAGCCAAGAGGTAATAAAATGAGGTCATAGATC 1200

1201 ACAAATAAGTTCATTTTTAATGGTTATTAACCTGGGGAGATAGGATAACTCCCCTTTAACAGTTCCCTTGAGACCTATTG 1280

1281 TACAGGTCACTAAATCTCATCTCGGCCACAACAAGAAGTGCACATCCTACGAGGGAGCGGCTGCCAGGAACGATAGTGAC 1360

1361 CGGCAGATGGAGGCGTCCTGAGCTACTACGCCGACCCAGAGGCCGGGGTTCACGAAGGGAGCGACTACCGGAAGGAGGGC 1440

1441 AGCTGGAGGTCGAGCTCAAGGCTCCCGCCTGATGCCTCCCGGAGGAGACACGTCAGACTCAGCCACACCGGTCACCCCCA 1520

1521 GGTGGCTGTAAGTCTGGTCCATCGCCTCCAACCCAAGAGGGAGACATCGTCTCCTCTCCAAGACCCACCAGTGTCCGAGT 1600

PBS

1601 GGGTAAACTGGGGGAGGAGCCACAAAGGATTT**CATGGTGCCCAACGTGGGGC**GTGGCCTAAACTAGGACCGCCCCCTGAG 1680

1681 AGAAGGGTTGGACCACAGGAGTGGCCCAGCTTGTCTACAAGAGCAGAAAATGAGAATTGACTGCTCAGTGTGCTAAAAGA 1760

1761 GAGCCAACAGCACACATCCTTCACAACACCCACTCGAGGAGTGGGAGAAGGAGAGGACCCTGCAGCAGGAGCTGCTGAAA 1840

1841 TGAACACAGCAAGCTCAGGGTAAGTAGCTACAATCGTCGAGACCCCATCCCGGTCCTGTAACGCGTGCAGGAGGCTTTGA 1920

Gag ORF

1921 GGATCGACAACTCCAATCAAGACAAAGAAGCACAATGGCTGCTGTAGCCTTGCCAGAGAATGAGGAGAAAGCGGGGACAT 2000

M A A V A L P E N E E K A G T S

2001 CACAAGAAACGGCCCCCGACCCAGAGCCCAGACCAGCCCCGCCATGCATAGTTATGCTCTATGGCAGAGGGACCAAGGAG 2080

Q E T A P D P E P R P A P P C I V M L Y G R G T K E

2081 TGGCACCCCACCGCTAAGGCGCTCTTCATGTCACATTCCACAAAGACAGACCCAGTGCAAGCCCTGGCTGAACTGGAAGA 2160

W H P T A K A L F M S H S T K T D P V Q A L A E L E D

2161 CCACATGACCAAAAACACATTCTTCTTTGGGCCCACCCTGGTGGATGGGGCCATGCTAGCCGTGCTGCTTCATCCACCAG 2240

H M T K N T F F F G P T L V D G A M L A V L L H P P E

2241 AAACCAAAAAGAAGGTTCAGAAGGAGGAATGGGTCAGATGGTGGCTGACAACAGTAAAAGGATGGAAAACCGACGTGGTG 2320

T K K K V Q K E E W V R W W L T T V K G W K T D V V

2321 AAGCTGGTAAGCCCGTCAGCAGAGTATGACCCCCTGGTAAAATTGGTTGCCAGGGAACAGTTCATCAACTGCATAGTGGC 2400

K L V S P S A E Y D P L V K L V A R E Q F I N C I V A

2401 CGATCCAGCATCCAAACCTGAGAAGAAATATAAGTGTCTGGAGCAGCCTCTGTATGAACTCAGGTTATGGAAAAACATGT 2480

D P A S K P E K K Y K C L E Q P L Y E L R L W K N M L

2481 TGAAGTACAAGGTCAAAAATGAGTATGAGGATGAGACCAGTGTGGTCGGAGATCCCGACACCAACACTGACCACGAAGAA 2560

K Y K V K N E Y E D E T S V V G D P D T N T D H E E

2561 TCAGGCACGGAGACTGATGATGAGGATGAGGATGATAAGAAAGGAAAACAAGATGATGAAGATTCTTTCGATTGGAAAGA 2640

S G T E T D D E D E D D K K G K Q D D E D S F D W K D

2641 CGGAGATTCTTTTGATGGTAAGGAAAAGGATGATGATGATCGATCCCACTTTCGCAAAGAGATAGGAGAGCTGACTCAGG 2720

G D S F D G K E K D D D D R S H F R K E I G E L T Q A

2721 CTTTGCAAGATATAAAAACGTACCTGGGTCAAACCCCCAAGTCAGAACCAAAAAATGATGGAAACCAGAACACTGTGAAG 2800

L Q D I K T Y L G Q T P K S E P K N D G N Q N T V K

2801 GCTTTGACTAAGCAGTTTGAAAACACCACCCTCAATGAAGGTGACAAAAAACATCGGAAATGTGACCGCAAACGGCGTCA 2880

A L T K Q F E N T T L N E G D K K H R K C D R K R R H

2881 CATAGAACCAGGGATACCGGGGCGAGGCCCGTTAGGCCCTCTCTCTGAAGAGACCCTGAAGATTCGAATGCAGAATGGTA 2960

I E P G I P G R G P L G P L S E E T L K I R M Q N G I

2961 TAGTCCTGCATCAAACTGTGGGAGACCCATGGGCCATCTCAAGTGATGGACTGCTGATATTCACCGACCACAACTACAAA 3040

V L H Q T V G D P W A I S S D G L L I F T D H N Y K

3041 AGTTACAATGCAAAATTCAGAAAAGCCCTGATAAAAAATGCGGGGCATGAATACCAGGAAGAGGTAAGGGAGTTAAGAGA 3120

S Y N A K F R K A L I K N A G H E Y Q E E V R E L R E

3121 AACCCACAAAAAAGATCCCGATCGACCCCAAATGACTAGTGGAGCCGGACTGGCCCACTATGGAATCATTCATGTCCCAG 3200

T H K K D P D R P Q M T S G A G L A H Y G I I H V P V

3201 TACTTGCATATAAGAAAGGTGATGACATGGAAAACTATGTCCGCCTAATGATGAAAAAATTGGAACGAGGTTTGTATCAC 3280

L A Y K K G D D M E N Y V R L M M K K L E R G L Y H

3281 ACGGTACATCAAGGACTGAGGAGAGTCGTCATCAGCACCTGTGGTTTGAGGTCTGGAGATCTCCCATGGGACATGGCAGA 3360

T V H Q G L R R V V I S T C G L R S G D L P W D M A E

3361 AAAGGTGGCAGTGAGAGGCTCAGCGCACTTCGCTCAGAACCTCAATGTGCCCACAGACCTGAAAGAAATCGTCGTGGTCA 3440

K V A V R G S A H F A Q N L N V P T D L K E I V V V T

3441 CAGCAGAAAAGCTGAAAAGTGAAAGGCTGAATAAGGCCATGCCAATGATACCCATAAAAACTGAGCCCATGCAGGGGGAG 3520

A E K L K S E R L N K A M P M I P I K T E P M Q G E

3521 GGGAAGAAGGCCCCCGTCAAAGAGACCCCCATCAAAGATACCCCGCCCAAAAAGACTGAACAGGGTCTCAAAGCTTCAGA 3600

G K K A P V K E T P I K D T P P K K T E Q G L K A S D

3601 CACAGAAAGAGATAAACCATTGGAAGGACCTGTGTGCAGCACCCAAAACAAGAGGACATTGAAGAACTCGATTTGGTCAG 3680

T E R D K P L E G P V C S T Q N K R T L K N S I W S E

3681 AGGAGGAGAAACAGGACCTAATTCGACACTTCAACAGGTGTGACACCAGCTCTGGAAACGAGAGCTCAGAAGAGGAAGAA 3760

E E K Q D L I R H F N R C D T S S G N E S S E E E E

3761 CCGGCGAACCCCCCCCGGCCTGTTGAGCGCCCACAAACCCCCGTACCCCATGAAAGCTCAGAAGAAGACAACGATGACTC 3840

P A N P P R P V E R P Q T P V P H E S S E E D N D D S

3841 CAGCATGGGGTCTGCTGCAGCACAAGGTCAAGGTACCCCTAAACAACTAAGGGTCGGACAAAGAAAAGGAGAAAAGAAGC 3920

S M G S A A A Q G Q G T P K Q L R V G Q R K G E K K Q

3921 AGGTGGAACTGGTGGATGTCCTCCGGGTGTCAGAGAGAATGGCACGAGAACCCAGGTGGGGACCAATCCAGACCAGTGAT 4000

V E L V D V L R V S E R M A R E P R W G P I Q T S D

4001 GGAAGAAGAACCTATGTGCCTGAAGCAGATCAGAGGGACTTCCCAATACCCCCAGCGTGGGCTGAAAAGATGGAGGAACA 4080

G R R T Y V P E A D Q R D F P I P P A W A E K M E E Q

4081 ACTGGAAATAAAGGTAAAGGCCAAAGTGGGGGAATGGGCTAACATAATAATGATGCCATCTTATCCCACTCTAGCTGCAC 4160

L E I K V K A K V G E W A N I I M M P S Y P T L A A H

4161 ACAAAAGATTGACAGCTAACTTCAGGCAAGCATATGTGGCCATTGCCCACGATGTGATGCTGAAAAAGATGAAGAGAGCA 4240

K R L T A N F R Q A Y V A I A H D V M L K K M K R A

4241 GCAAACAAGTTCGTGAGGGACATGGTGTATGAAGAGGAAGATGGGATGCTCTCGGACGAACCAGTACCGCCAGTAACGGC 4320

A N K F V R D M V Y E E E D G M L S D E P V P P V T A

4321 CGCAGGTGAAAAGGACTCATCAGGAAGGCAGGAGTGGAAATCAATTAAAGATGTGAGCACCAACGCAGATGAGTTCCGTG 4400

A G E K D S S G R Q E W K S I K D V S T N A D E F R E

4401 AGTATAAGAAAATAAAAACCCTGGTAAGAGAACCAGGTGCAGGCGAAGGAAAAGAAAGCTATCTAAAAGATGTCTGGCCC 4480

Y K K I K T L V R E P G A G E G K E S Y L K D V W P

4481 CTCGTCGTGAACTCCACTGACTGCGAGGCCGCAAAGCGCCTGTGGCTGAGTCATATCACAGGCCATCCCATCGACAGAAA 4560

L V V N S T D C E A A K R L W L S H I T G H P I D R K

4561 AGACTCGGCTCAGGACTACTATGAAAAATTTGTACCTGAAGATGAAAATGATGAGGATGCCCACATTGACAGGGCTCGAC 4640

D S A Q D Y Y E K F V P E D E N D E D A H I D R A R L

4641 TCAGACTGGAACAAGGACAAAAATTAACCCCCATCTGGCATCAGCTGAAACAAAACATCGCCCCAACCAGGTATGTGAAG 4720

R L E Q G Q K L T P I W H Q L K Q N I A P T R Y V K

4721 GCCCTAAGGATGGTGACAAAGGGACGAAGGGGTGACATAACTTTTGCCACCATGCCTATCCCAACCACCACAGTGGCACA 4800

A L R M V T K G R R G D I T F A T M P I P T T T V A Q

4801 AATGGATGCAGCAGTAAGGAAGTATGACCTGGAACTGCTCTATTGGGATGAAAGAAGAAAAAAGGAGTCAACCCCGAAGC 4880

M D A A V R K Y D L E L L Y W D E R R K K E S T P K P

4881 CACCAAAAGTCCCCACCAAAGCGGCCCTGAAGGAGCCGGCCAATCCCCAGGGTAAATTTCATCAACCAACCCCTCAGCAG 4960

P K V P T K A A L K E P A N P Q G K F H Q P T P Q Q

4961 AGGGACAAAGGCCCCAGACAGCAGTACCAGCCAAACAACCAAGGACAACAATATAGGCCCCCGCCACAACGACAGCAAGG 5040

R D K G P R Q Q Y Q P N N Q G Q Q Y R P P P Q R Q Q G

5041 ACCGGAAAGAGGAGGTCCGGCGACTCGGCCTGCTCAAGGCCCCAAAAGAGCCCCTTACTTGCCCCCAGAAGAGTACAACA 5120

P E R G G P A T R P A Q G P K R A P Y L P P E E Y N K

Gag ORF end **Pol ORF**

5121 AATTGACCCCAGAACAAAAAGATGCCCTCCGTCAGGCCAGGGAACAAGTGGCCGGAGGGCCGCCAAAATGATGGACCCCA 5200

L T P E Q K D A L R Q A R E Q V A G G P P K *

M M D P R

5201 GGTCCCGGGTCACCCTGGAAAACACCTACTGGAATGGGGACCACATCTTCACCAATGTAGATGGGACTCTCTTCCTGGTG 5280

S R V T L E N T Y W N G D H I F T N V D G T L F L V

5281 GATACAGGGGCAGAGGTATCCATGACCCGGAGAGACCTAGAGACTGAAGGGTACCTGTATGTTCAACTGGCTAGTGGAGC 5360

D T G A E V S M T R R D L E T E G Y L Y V Q L A S G A

5361 AATGGAGGAAATGCCATATGGAATTTGGAAAAATGTGATATGGTTAAAAGGACCTTATAATTTGGTAACGGTCAAAGACT 5440

M E E M P Y G I W K N V I W L K G P Y N L V T V K D L

5441 TGAAGATCCTGCATGACCCCGAGGAAGCTAGGAAATCAGAATGGAAAAGATTGGAGATGTTAAAATCAAAATCAATAAAA 5520

K I L H D P E E A R K S E W K R L E M L K S K S I K

5521 ATAAGCCCCTCAGCCACCACAACGTGGTACAAACCAGTGGATGTCCCTGAAGTAACAAAACAAAAGTTGGAAGAAAGTGA 5600

I S P S A T T T W Y K P V D V P E V T K Q K L E E S D

5601 TTTGTCCCCAGAAGGAAAGATTAAATTAAAACAAATAATTGAATCAGCCAGAATTGCTCGCTTCAAAAATGACTGCGGAG 5680

Aspartic protease

L S P E G K I K L K Q I I E S A R I A R F K N D C G D

5681 ACTTGGGCACAAAATTTTGCCATCATATCATAGGAGGGGTACACCCACCTGTAAAACAGTACCCTCTCCACCCAGAAGCA 5760

L G T K F C H H I I G G V H P P V K Q Y P L H P E A

5761 GTGGCAGAAATGGACAAAATTGTAAAAGAACTGCATGCACTGGGAATCATCAGAGAAGAGCCAAATCCCCTCACAAATAG 5840

V A E M D K I V K E L H A L G I I R E E P N P L T N S

5841 CCCTATTCAGGCTGTGAAGAAACCTGAATCGGCGGGAGGAGGCTGGAGGCCAGTGATCAATTTCAAGGCCCTAAACCGGA 5920

P I Q A V K K P E S A G G G W R P V I N F K A L N R R

5921 GAACTGTCGCAAATCGAGCCAGCCTCATCAATCCCCAAGGAGCATTAAAAACACTGAGAGTTAAAAAGTACAAATCTTGC 6000

T V A N R A S L I N P Q G A L K T L R V K K Y K S C

6001 ATCGACCTGGCTAACGGGTTTTTCTCACTCAGGTTAGCCCGTGAATCACAAGGAAAAACAGCTTTCACCCACAAAGGTAA 6080

I D L A N G F F S L R L A R E S Q G K T A F T H K G K

6081 AAGCTATGTGTGGCAAAGACTGCCACAGGGATACAGAAACTCACCAAATGTATTCCAATCAGCCGTCCTGGAAATACTTG 6160

Reverse transcriptase domain

S Y V W Q R L P Q G Y R N S P N V F Q S A V L E I L E

6161 AAGATGTTGGAGCTTCAGTGTACATCGATGACGTTTTCATTGCAGATGACACTGAAGAAGAGCACCTTAAACGTCTGGAA 6240

D V G A S V Y I D D V F I A D D T E E E H L K R L E

6241 GAAGTGGTTAAACGGATCTCTGCAGCAGGCCTCAAGCTAAACCTGGCGAAATGCCAGTTTGGCCAGTTTAAAGTCAACTA 6320

E V V K R I S A A G L K L N L A K C Q F G Q F K V N Y

6321 CCTGGGGTTCCAAGTCACCACAGACTTGGGCTTATCAGACGGTTACAGAGAGAAAATTCAACAAATACAACCCCCCAGAT 6400

L G F Q V T T D L G L S D G Y R E K I Q Q I Q P P R C

6401 GTGAAAATGATCTACAGAAAATCTTAGGCCTGTGCAACTATGTGAGAGATCACGTACCAAACTACCAGAAGTATGCAAAA 6480

E N D L Q K I L G L C N Y V R D H V P N Y Q K Y A K

6481 CCCTTGTATGCATGCCTGAAAAAAGAGGCTACGACAGAAGGCCGCAAGGGAAAAGCATGGTCATGGACACCCAAAAACCA 6560

P L Y A C L K K E A T T E G R K G K A W S W T P K N Q

6561 AGACGATCTTGACAAGCTGAAAGAAGAGATCCAGAAAGCTGTCAGACTTGAACCTAGAAACCTGACCTCTAGGCTGGTGG 6640

D D L D K L K E E I Q K A V R L E P R N L T S R L V A

6641 CAGAAATATTCTGCGAGAACGAAGACGCCATGGTCAAAGTGAGTAATGATCAAGGAGGCCTAATCACATTGTGGAGCACA 6720

E I F C E N E D A M V K V S N D Q G G L I T L W S T

6721 ACGCTGACATCAGTGGAGAAGAAATACCCCCAAGAAGAGAAGGAACTGGCAGTGTTAGCAAAATATTGGGGGTCACTAAA 6800

T L T S V E K K Y P Q E E K E L A V L A K Y W G S L K

6801 AGATCTGGCCCAAGGTCAACCCATCAAAGTCACGACTCAAAGTCAGGTACACAAATACCTGAGAAAAGGCACCATCGAGA 6880

D L A Q G Q P I K V T T Q S Q V H K Y L R K G T I E S

6881 GCACAAGAGCAACTAACACCAGATGGGGCCGATGGGAAGACATTTTGCTGGATCCAGACCTAGAAATAGGCCCGGCACAG 6960

T R A T N T R W G R W E D I L L D P D L E I G P A Q

6961 TTACAGCAAAAGAAGCCTACTGCCAGTCAGACTAAGGATAAAACATATGACTGGGTGCTCTTCACAGATGGATCACGAAA 7040

L Q Q K K P T A S Q T K D K T Y D W V L F T D G S R K

7041 AGGCCCTGATGAAACGGCATATTGGGGTTACATCTTGACACAAAATGGGAAAGAACAACACAGAGCCAGAGGAAAGGTGA 7120

G P D E T A Y W G Y I L T Q N G K E Q H R A R G K V S

7121 GTGGAAGCGCCCAAGCTGGAGAAGTGACGGCGGTTTTGGAAGGGTTGCTGGAACTCACCAAGAGGAAAATCAAGGCCGCA 7200

G S A Q A G E V T A V L E G L L E L T K R K I K A A

7201 AGGGTAGTAACTGACAGTTACTATTGTGCACAGGCCTTGAAAGAAGACTTGTCCATCTGGATTGAGAATGGATTTGAGTC 7280

RNaseH domain

R V V T D S Y Y C A Q A L K E D L S I W I E N G F E S

7281 AGCCAGAGGAAAAACTGTTGCCCACAAAGAACTTTGGGAAAAAATAGCCGAGTTAAGGACACAACTCGACTTAGACGTGG 7360

A R G K T V A H K E L W E K I A E L R T Q L D L D V E

7361 AGCACCAAAAAGCGCACACAAAAGAAGGATCTTACTGGGAAGGAAACGACGAAGTGGACAGGTTTGTCCAACAACGGAAA 7440

H Q K A H T K E G S Y W E G N D E V D R F V Q Q R K

7441 ATTGTCTTGATTGGAGCAGAAAAATGGAATGTTACTCCTAAAGGAAAGGAGGTTCCAAAAGAGTACGTGGCGGAGGTAGT 7520

I V L I G A E K W N V T P K G K E V P K E Y V A E V V

7521 GGTGACCATGCATGAGGCTCTAGGACATGCTGGGCTGCTACCCACACGTAGAGGACTGGTAAAGCAGAACTTATGGATTC 7600

V T M H E A L G H A G L L P T R R G L V K Q N L W I P

```
7601 CTATGAAGCAGATTCAGCAAGTACTCAAACGCTGCACGGTATGTGGGAAATACAATGCAGGAAGAAGAGGAAAAAGAACT 7680
      M  K  Q  I  Q  Q  V  L  K  R  C  T  V  C  G  K  Y  N  A  G  R  R  G  K  R  T

7681 GAGGGCCTCACCATTAAAAGCACAATCCCATGGGGCTCAATATGCATGGACGTAGCTGGACCCATGGGGGTCACAGGGAC 7760
     E  G  L  T  I  K  S  T  I  P  W  G  S  I  C  M  D  V  A  G  P  M  G  V  T  G  T

7761 TAAAGGTGAGAAATATCTTATTGTCCTTGTAGACTCAATGTCTGGCTTTGTGGTGGTGAGACCTGTCAGGAAAGCCAACG 7840
      K  G  E  K  Y  L  I  V  L  V  D  S  M  S  G  F  V  V  V  R  P  V  R  K  A  N  G

7841 GGAATAGTGTTGTCTCCATGTTAGACTACACATGTGCCTGTTTAGGCATTCCTCGAGAGCTACGAACCGACAATGGAACG 7920
                                        Integrase core domain
      N  S  V  V  S  M  L  D  Y  T  C  A  C  L  G  I  P  R  E  L  R  T  D  N  G  T

7921 CATTTTAAAAATGTGCAAGTGAACAGGTGGTGTGAGCAACATGGGGTGATGAGGGTATATTCACCGCCATACACGCCACA 8000
     H  F  K  N  V  Q  V  N  R  W  C  E  Q  H  G  V  M  R  V  Y  S  P  P  Y  T  P  Q

8001 GGCCAACGGCGTTGCTGAATGTACCATCGGTTTGGTCAAAAACTGGCTAGCTAAAAATGCTAACTCAAAGGAATGGAGCC 8080
      A  N  G  V  A  E  C  T  I  G  L  V  K  N  W  L  A  K  N  A  N  S  K  E  W  S  L

8081 TCAAGTCGGTCGACATAGGCCGAGCCCTCAACGACCGGGACCGAGCAGGAAGGCCGTCCCCTTCTGACGAACTAAACCAG 8160
      K  S  V  D  I  G  R  A  L  N  D  R  D  R  A  G  R  P  S  P  S  D  E  L  N  Q

8161 CGTCCCTTTGTCACTGAGGAAACAGGGAGGAGTCAGGAGGCGGACCCAGCCCCCTTAGAGGCATGCCCATTCGCTGTGGG 8240
     R  P  F  V  T  E  E  T  G  R  S  Q  E  A  D  P  A  P  L  E  A  C  P  F  A  V  G

8241 ACAGAAGGTGTGGCTTAAAGCCAGAGACCAACCCAGTACGGGGGCAGTAAAGCCAAAGTATGAAGAGGTTGACACTGTGA 8320
      Q  K  V  W  L  K  A  R  D  Q  P  S  T  G  A  V  K  P  K  Y  E  E  V  D  T  V  T

8321 CTAAAGTGTTGGACCAGAACACAGTGCTGCTGAAGAAGAAAGGAATCCAAGGAGTTGAGCAGCTGAAACCAGCCTTAGGT 8400
      K  V  L  D  Q  N  T  V  L  L  K  K  K  G  I  Q  G  V  E  Q  L  K  P  A  L  G

Pol ORF end          Acc3 ORF
8401 TAAGGAACCAGAAATGAACATGGCCATGGACCTCAGCAACTCTCCACCGCCCTACATTCGACTGGCCACGATTAACGGGT 8480
     *
                  M  N  M  A  M  D  L  S  N  S  P  P  P  Y  I  R  L  A  T  I  N  G  L

8481 TGGTAATCATGAGAAGAACCACATTGGGTATTTGGGTTGGCAGAGCCCTAAAGACCCTAGAATATGCAAAAATTGGTTAT 8560
      V  I  M  R  R  T  T  L  G  I  W  V  G  R  A  L  K  T  L  E  Y  A  K  I  G  Y

8561 CTGTCACCGGATCCAGAAACAGTGGAATGGAACCACTGTAATGTGGGATGTGTGGTTTGCCTGCAGGCAGGAAACTTTGA 8640
     L  S  P  D  P  E  T  V  E  W  N  H  C  N  V  G  C  V  V  C  L  Q  A  G  N  F  E

8641 GACCGCCTGGGTTGGACCCGGCCACAAGAAACCACTCTCCCCTCCACGGTCAAACGTTGACCTCATTGGAATCAGGAGAA 8720
      T  A  W  V  G  P  G  H  K  K  P  L  S  P  P  R  S  N  V  D  L  I  G  I  R  R  S

8721 GCCCAGTGAAGGTGCTGGATGCCTTCATCTGTGGAACATGCAGAGTTGACACTCTGCCCAGAATTACCTTTGTCGTCCAA 8800
      P  V  K  V  L  D  A  F  I  C  G  T  C  R  V  D  T  L  P  R  I  T  F  V  V  Q

8801 TGGGACCGCAAAGGAGTGCTGCCCGAGGGATGTGGTTGTTCGTGGGATGGTCACCAACTGGAGTGGTGTCCCTCATGTTC 8880
```

```
     W  D  R  K  G  V  L  P  E  G  C  G  C  S  W  D  G  H  Q  L  E  W  C  P  S  C  S
8881 ATCCCTAGGCTGCCATGGAACCCTGGTCCAAGCGGCTGAAGCTCGTGGATGGCAGGTGCGGCGGCTCTTTTACCCCCTCA 8960
      S  L  G  C  H  G  T  L  V  Q  A  A  E  A  R  G  W  Q  V  R  R  L  F  Y  P  L  R
8961 GGTACTTAACACCAGATGAGACCGCTGACGCTGTTGCATCCTCCATGGGCCTCGGTCCCCTTCTCTACCCTGAACTCTCC 9040
       Y  L  T  P  D  E  T  A  D  A  V  A  S  S  M  G  L  G  P  L  L  Y  P  E  L  S
9041 TGTGATCCCTCTGCACCCCCCTTGGATCCTGTCACTGATGAGAAGGAGGAGGGAGACACAGAGGCACCCGAGGACCTCCG 9120
     C  D  P  S  A  P  P  L  D  P  V  T  D  E  K  E  E  G  D  T  E  A  P  E  D  L  R
9121 CTGTGACCTGTTGGACGAAACCATCGACAAATCTTGGCCCTTTCAAGCGGCCGGGATTCGTGCAGGAAGGCCCTTGCTGG 9200
      C  D  L  L  D  E  T  I  D  K  S  W  P  F  Q  A  A  G  I  R  A  G  R  P  L  L  G
9201 GACTACATGAGTACCCTTTGGTTTTCAGGGTTTATAGAGAACCTTTAACATTGGCCCTCAGACCTGACGACAGGCCAGAT 9280
       L  H  E  Y  P  L  V  F  R  V  Y  R  E  P  L  T  L  A  L  R  P  D  D  R  P  D
9281 GATGTAAAAGCGACTGCACAAGAAGGCGGCTGGGAAGGACCCTGGGAGCTCACCACATGGGCCTCCCTAATAAAAGAGGT 9360
     D  V  K  A  T  A  Q  E  G  G  W  E  G  P  W  E  L  T  T  W  A  S  L  I  K  E  V
9361 TATCCAGAATCAGTTCATCCCGCTGATAGACCTCCCACCTATCCCTCGTGAGGAGAGGGTCAGAATGTCTGACCTATACT 9440
      I  Q  N  Q  F  I  P  L  I  D  L  P  P  I  P  R  E  E  R  V  R  M  S  D  L  Y  Y
9441 ACTACACCACCAACATTGATGCTTGGTGTCCGAGAGGAGCTCGCCCCAAGCAAAAGGTTGCAACATACACTGTCACCTCT 9520
       Y  T  T  N  I  D  A  W  C  P  R  G  A  R  P  K  Q  K  V  A  T  Y  T  V  T  S
9521 GAGGCGTGGAATCGGCAAGGAGTTCTACAGATCCCCGGGACCGTGGCTCAGAGAACTCCAATTCCCATCCGCCCTGATTC 9600
     E  A  W  N  R  Q  G  V  L  Q  I  P  G  T  V  A  Q  R  T  P  I  P  I  R  P  D  S
9601 CACTTACAAAGTGCAGGTCGCAGTTCAAATGGTGATTGTCCCCCTTAAAGGGGTTTATTCCATTGGAGCCACGATCACTG 9680
      T  Y  K  V  Q  V  A  V  Q  M  V  I  V  P  L  K  G  V  Y  S  I  G  A  T  I  T  V
9681 TTCTTGGACGACATGGTGAGGTGGTCAACACAGATGGGGCCCCTGTCCCGCTCCCTGAGGCTCCCGCCCCACCACCTCGC 9760
       L  G  R  H  G  E  V  V  N  T  D  G  A  P  V  P  L  P  E  A  P  A  P  P  P  R
                                                                      Acc3 ORF end
9761 CGCCACTCTGGCTTGCTCTTTAGAGCCCTAACCCGCGTCCCCTCGCGGTTGCGCAGGGTGAAGAGCGAGACAGAATAAAT 9840
     R  H  S  G  L  L  F  R  A  L  T  R  V  P  S  R  L  R  R  V  K  S  E  T  E  *
9841 TAATGGTGCTCCAGTGAGATGTACGGTTTCCCCTTCATTCGACTTATGATATATTTTTCTCTTTCCTTGGGATTGGCTGT 9920
9921 GGGATGATTGACCTCAATAATCCCAGGAGAGGGGGTGGGGCCCTGAAGATGAGGAAGGACTGATTGTAACCACACCCCAC 10000
10001 CAGAGGAGAAACCCCCCCCCTCATAAAGATAAAAAGCCTGTCTGTGAGCTGCACAGTAACAGAAAGAAGATGGAGAGGA 10080
                                               Env ORF
10081 TCACCTACATGTCACAGACCCAGGAAGACCCAGAGGACAATGACAGGGAAACCGACCTCACAAAAAAACCCCCTTCCCAA 10160
                                              M  T  G  K  P  T  S  Q  K  N  P  L  P  K
10161 ACACAGCTGCGGCTGCAGCTGTAAAAAGAAGCCATGTCTGCAGACTGGTTTAAGGCTTTTAGCTTTGTTTCTTGCTGTTG 10240
       H  S  C  G  C  S  C  K  K  K  P  C  L  Q  T  G  L  R  L  L  A  L  F  L  A  V  A
10241 CTTTAATTTTGTCAATTTTGTACCAAGCAGGATACCAACCATGGAAAGTCACTCATGCCAGAGTGTTTCATGCCAACACA 10320
       L  I  L  S  I  L  Y  Q  A  G  Y  Q  P  W  K  V  T  H  A  R  V  F  H  A  N  T
```

10321 AAATATGGAAATTTGACTTGCTGGAACACCAGCGCCCGCAACCTCTTCATGCCCTGGGATGCAAACCAAACTTCTGAGAA 10400

K Y G N L T C W N T S A R N L F M P W D A N Q T S E N

10401 TCAAACTGACTCCTGGAACAGGATAAAAAGAATGGGTTATGAAGCATGGCGAGACAACCTGTATGATTGGACTGCCTGGA 10480

Q T D S W N R I K R M G Y E A W R D N L Y D W T A W T

10481 CAGATGAACTGGCACGACAAAACTTCTCGACGGACCACAGATGGACAATCTCAAATCTACTGGCAACTAAAGATGGATTG 10560

D E L A R Q N F S T D H R W T I S N L L A T K D G L

10561 GTCGTGTGGGTTAAGGTATTAGAAGATCATGAGACTAAAACGACGAAGTGCAACTGGATACAGCCAGAGGGAGAACCACC 10640

V V W V K V L E D H E T K T T K C N W I Q P E G E P P

10641 CGAATGGCACTGTGATTGGGGATGCAGGCCCCTGCTGGGCCTAGCGCACCAAGGGTACTGGCAATATGAAGCTGTATTTC 10720

E W H C D W G C R P L L G L A H Q G Y W Q Y E A V F P

10721 CAGCACCCCACAAACACATATTCTGCCCGGCCATATTTCCAAGATGGGAATTTTGGGGACACTATGACCTCAAATGTGAC 10800

A P H K H I F C P A I F P R W E F W G H Y D L K C D

10801 AAAATCCCAGTCACAAACAGAACCAGAATTAGACCACAAAGAAAGCACCCGACAGCCTATAAAAAGATGCCCAGAAGGTC 10880

K I P V T N R T R I R P Q R K H P T A Y K K M P R R S

10881 CATACGCCCCAATAAAACTGGCAATTATACTGTAACGGTGCAAACCTATAATAATGAGACTACCAATGCAACGGGAATTG 10960

I R P N K T G N Y T V T V Q T Y N N E T T N A T G I E

10961 AAGCACAAACCACTCCGCCCCCAGGTACCACTCCGGCCCCAGCCCAGGACCCAGAGAAATACAACACCGTGGATGAATGC 11040

A Q T T P P P G T T P A P A Q D P E K Y N T V D E C

11041 TTAGCAGACATCACACGCCTCGACTCCTGCATCTGGAACACGTGGGTAAAATGTAAAGGCCAAAACTTTATGCCCCTGGG 11120

L A D I T R L D S C I W N T W V K C K G Q N F M P L G

11121 CCCGCTGTGGTTCCAGACTTACTATTACACTAGCTGGAGATCAATTTTCAGTCCTACCAGGGCTATGCATGAGGAATGGC 11200

P L W F Q T Y Y Y T S W R S I F S P T R A M H E E W L

11201 TAAACTATACCTTCCCATGGATATGGATCAAAGAGGATGTTGTTCACTATGAAGGAGATTTCCCTGAAGGGTCTACTAGG 11280

N Y T F P W I W I K E D V V H Y E G D F P E G S T R

11281 AACCGTGGAGGGCTTAAGGGCCCTTACCGGTCACACATGTATGTCCAAAAATTGCCCACTGTCCAAGAGTTAAAATCAGT 11360

N R G G L K G P Y R S H M Y V Q K L P T V Q E L K S V

11361 ACCCAAAGGAGATTTTGACAAAATAGACAAATGTGTTGCTAGAAGAATTTTCCTCAGCCTCAATGATTCTGAATGGGAAG 11440

P K G D F D K I D K C V A R R I F L S L N D S E W E G

11441 GACATGGGGCCTCCCCTAGATGTAAACTGTGGCAGGTTGATATCTCCTTTGTGTCAACATGGAGGTATGACTGCACAGGC 11520

H G A S P R C K L W Q V D I S F V S T W R Y D C T G

11521 ATCCTGAGGTACAACAGCACCAAAGAACTGCTGCAAGGATGGGTTGAAGGACACAGCAGAGATAAAACCCGGACATGGTG 11600

I L R Y N S T K E L L Q G W V E G H S R D K T R T W W

11601 GGGGTTTCAAAGAGAAGAAATGGAACACTGGGTGATCCCCTGCCTGTCCCAATCCGACAATTATTGGGTAAAGTATCAGT 11680

G F Q R E E M E H W V I P C L S Q S D N Y W V K Y Q Y

11681 ATATTGCAACGGTGTATTCAAAAGAGCATGATATCTGGTCCTCAGCAATTTACAGACCAGACAGGTATGTCCGCCCCAGA 11760

I A T V Y S K E H D I W S S A I Y R P D R Y V R P R

11761 CCATGGAAATTGACCTGTATCAAAAGTGGGCACGCTTGCACAGTAAGTCTGGCACGAAGACCCTGCAGTGAACTACCAGG 11840

P W K L T C I K S G H A C T V S L A R R P C S E L P G

11841 CTGGGAAGGTTATTGTGGAAGTTTTTATGCAGACGATTATAATACTCATGAGGCTGCGACAGTATGGACCAAGACACGAC 11920

W E G Y C G S F Y A D D Y N T H E A A T V W T K T R P

11921 CAACAACTTATACAAAATTGATATCAAATGCGACCAACACATGGCGTCGAGCCATGGCCTGCGATCACTGTGTTAATGCC 12000

T T Y T K L I S N A T N T W R R A M A C D H C V N A

12001 ATCATAGGACCCCACATGAAGTCCCGGAAGAGTATTGCTGGTGAAATTATCCCCTCAGTTCCCGACACTATGATCACAAT 12080

I I G P H M K S R K S I A G E I I P S V P D T M I T I

12081 AGCTGAGAGTCACGCTTTCAGAAGAGGGCTGTGCCTAGGCCTTGAAGAGGAAGGATTTGAGTGGGTAGGGCCAAACCTAC 12160

A E S H A F R R G L C L G L E E E G F E W V G P N L L

12161 TCTACTCTAACCTCACATGCAAATACCCAGAAGAAGTTTGGAAGAAATGTGGCCCGGACTCCGACAAACACACCTGTGCA 12240

Y S N L T C K Y P E E V W K K C G P D S D K H T C A

12241 TGGTATGAAACCCTGCAAACAATGGCCCTCAGAGAGGTAAAAACCCTGGCAAAAGGGGCCATTGAAATTGTTGCCGAAGG 12320

W Y E T L Q T M A L R E V K T L A K G A I E I V A E G

12321 AGCAGGAGCAGTTGAGGAAGGTATGGTGGAGTTGTTGGAAAAGATTTGGCCATACTGCTTGACGGTCCTTGGCATCCTGA 12400

A G A V E E G M V E L L E K I W P Y C L T V L G I L I

12401 TTGCTGCTGTCTTGGCATGCCTGGCTCTAAGAGAATTGTGTCTCAGAGCCCTGCCCCCAAGGAGGGGTGAGTACAAGCCC 12480

A A V L A C L A L R E L C L R A L P P R R G E Y K P

Env ORF end

12481 CTTCCCCAAGGTAGAGAGTAACTGATCCTATATAAAGCTGAGAGCACATAGCCTCAGTACGCAGTATTGCTATCGCTTTC 12560

L P Q G R E *

12561 TTTAAACGCTGCAGAAACGCTATGAGAATTAAGATTGGAGTTAGAGACTGGGATTATGGGAGCATAAAATTGACGGTGGA 12640

12641 AGTAGATCCAACCCATCGAGAAGACCTCCTGACAGTTGAGTATTGCAATCTCCAGGATTATCGTTATCGTGTCTACCCCT 12720

12721 CAGTAGACACAAAAGAAGAGTTCCTGGATTTGTTCTGTCAGGCCTTGAATAGCTGGGCAAGTGTGGATTGGCATCGCCAG 12800

12801 CATGAATTTCGTGGCGAACATTTCATGAATTAGGAAAAATTGACCTCTGCCCAAAAAATGAAGATTATTATGGAAAAGAG 12880

12881 ACAATGGGGGCGAGGAGCTCCGAAGATCAGTGTGGAAATAGATCCAATCTACCCTGAAGAAATTCTAATTTTGGATTATC 12960

12961 ACGTTGGTCCTTATTGGTTCAACAGAAGATATCGAATGTCATCTAGGAGAAATTTGTTGGAAATGACGGCCGGGACAATG 13040

13041 AGAATAATCACCGCAAATCAAGAATCATTCCTCTATGAAATAAGAGGCCACCATTTTCAAAATTGACTGTAAGGCCAAAA 13120

13121 GAGGAGGGGACTCGAACCCCTCCCCTCCCAGAGGAGGAGTAACAGTACCAGATAAAATAGGAAGACCCGAAGCCAGAACA 13200

13201 ACACTTGAACCCACTCAGCAACGGAGGACGATGCACATCACGATCCAACAGAGTCATTTTCACCCTGACCCGAACTACAG 13280

13281 GATCAGAAACTTCAGAATATTTCTGGAGGTGAACCCATCTCGGGAGAGACACATCCTGACTGTGGAGCAGCCTAGGAAGA 13360

13361 TCCTTGGAGCAGCCCCAAGGGAGGCACTAAACTTACACTACGGTGATCAAGAATTTTTCACTTATAAACAGGTCCTCGAC 13440

```
                                                                                    Acc1 ORF
13441 GCAACATTAAAGCAAATGTTGGAATGGGCCGGACCACAAGCTATCCCCCCGTTCTACATTATCCCAAATGAACCTCCCTC 13520
                                                                        M  N  L  P  R

13521 GAACTAGTGGGCAGATCAAAAGAGGAGGAGCAACACAAGCTCCGCCCCTAAGGAGGGCGCTGGTTCCCTATAAAACAGGC 13600
        T  S  G  Q  I  K  R  G  G  A  T  Q  A  P  P  L  R  R  A  L  V  P  Y  K  T  G

13601 TGCGCTGAGACAAAAGAGGAACAACCCCACTGGACCTGGACGAAGACACAAGAAGAGGAACAACTGCAGCATACCATGGC 13680
      C  A  E  T  K  E  E  Q  P  H  W  T  W  T  K  T  Q  E  E  E  Q  L  Q  H  T  M  A

13681 TTCTACCCAGCTACCTACCACACCCGACGTAAGTCCCACTTTTAACTCATCCATGCAGCATACCCGCTCACCGAGACCCA 13760
       S  T  Q  L  P  T  T  P  D  V  S  P  T  F  N  S  S  M  Q  H  T  R  S  P  R  P  R

13761 GGGATCCTCCCTTGGCTGTATCTCTGGTTGAATACTATGCGATAATGTTTGCATGGTTCTGTTACTGCATGACGGGGAAA 13840
        D  P  P  L  A  V  S  L  V  E  Y  Y  A  I  M  F  A  W  F  C  Y  C  M  T  G  K

13841 TGGACTCTCAAGCAACCGTTGAGAATTCTTATCCTGGCCATGTCAATGGGTCAGGTTGCAGTTGGCCGCCTTTTAGGCCG 13920
      W  T  L  K  Q  P  L  R  I  L  I  L  A  M  S  M  G  Q  V  A  V  G  R  L  L  G  R

13921 ACAAGAAGTCGCACTCTCCCTGTTCGTCTCCTACATCCTGGGGAGAGCGAAGCCACTGCAACCCCGGGCGGCTGCTTTAC 14000
       Q  E  V  A  L  S  L  F  V  S  Y  I  L  G  R  A  K  P  L  Q  P  R  A  A  A  L  L

14001 TTCTCTTATGGTTGCTTATTGAGTTCGTGGTAATAGGCATTGAATGGGTCAGACGAGCACCATTCTCCCGCTTTGCCCCA 14080
        L  L  W  L  L  I  E  F  V  V  I  G  I  E  W  V  R  R  A  P  F  S  R  F  A  P

14081 GAGTACATCATCCTGGCTGTCTGGGAAGGTATGCTGGCCATGAGTGTCATCTTTGTCATCGGTCGTGAAACCTGCCGCAT 14160
      E  Y  I  I  L  A  V  W  E  G  M  L  A  M  S  V  I  F  V  I  G  R  E  T  C  R  I

14161 AACATGTGGGAAGGTGGCCTGGTTCCTTCGGTTCCTCCGCCTTGCTCTGTGGGGCATAGCCTGGGGCGTTTATTCAACTT 14240
       T  C  G  K  V  A  W  F  L  R  F  L  R  L  A  L  W  G  I  A  W  G  V  Y  S  T  L

14241 TATATTGGATGGGACTGATCAGCGAGTTGTCGATCCTTGCCTGGTTCATGACCTATGCTGCCCTCCTCCCTGTGGCTGTC 14320
        Y  W  M  G  L  I  S  E  L  S  I  L  A  W  F  M  T  Y  A  A  L  L  P  V  A  V

14321 TCAGCTGAACAAGCCTCACTCGAACCACCTCCTCTTCCTCCAAGAAACGCGCCGATCACCACCCAGCCTAGTAGAGCTCG 14400
      S  A  E  Q  A  S  L  E  P  P  P  L  P  P  R  N  A  P  I  T  T  Q  P  S  R  A  R

14401 GCCGCTCCAGCGCTGGGTTAATCCTCCCAATTTGCCTCCCTTCGATGCTTCTCTCTACCATAACTGCCCCCATTCTCAAG 14480
       P  L  Q  R  W  V  N  P  P  N  L  P  P  F  D  A  S  L  Y  H  N  C  P  H  S  Q  D
Acc1 ORF end
14481 ATGATTAATTTTTTTGCTTTTTGCTGCTTTTAAGCCACATTTTTATGATTTTTATAATTTTTAAAGACATTTTTGATTTT 14560
        D  *

14561 TGATTTTGTGTTTTACTACAACTTATTGTTGCGTCTGGCGCTTTAAGTTGTAAAATGTTTTATGATCACTCCTAACCTCC 14640

14641 TTCACCCCTGTGTTCTGACAGGTACAACATTGTGCAGACTGCGGGAAAGGCTTGAGGGAGTTCCCATACCTTTGGGAATA 14720

14721 CCTCGGGGCTGCGTGGTGCACTCCATGCTACGCCTCCTACTTTGGAATCCCGGTGGCTACGGCCATCATACTGAGAGATG 14800
                                                    Acc2 ORF
14801 GGATCCAAAGCTGCCCCCGAGCTGACTTCTCCCAAAGCCTCATGCTGCATAAGGATGGACAGATGGTCCACTGTACTCTA 14880
                                                M  L  H  K  D  G  Q  M  V  H  C  T  L
```

```
14881 CAGAACCCAAACCTTCCTGAACCCCTTGTACCTGTCAATGTTCACCAGGCCTGTACCATCCCCCTCATCCAGAAGACCTG 14960
      Q  N  P  N  L  P  E  P  L  V  P  V  N  V  H  Q  A  C  T  I  P  L  I  Q  K  T  W
14961 GAAATCTCTCTCCACTAACCGCAAGTATATCAGCCTTCATACCCCTGATTATGCTTTGGTGGATCTGTACATCAGCCAAC 15040
      K  S  L  S  T  N  R  K  Y  I  S  L  H  T  P  D  Y  A  L  V  D  L  Y  I  S  Q  P
15041 CTGCTGAAGGAATGAGTACCGTGCAACAGGCGGTGGCCGACCTGCCGCGCCAGCACATCATGCCAGGGCCATCTCCAGAT 15120
       A  E  G  M  S  T  V  Q  Q  A  V  A  D  L  P  R  Q  H  I  M  P  G  P  S  P  D
15121 TCTATGACTGATCCTGCTCCTCAGGACATGTCATCACTGCAAGATGCCCTCCATGACCCCGACCTTATTGCTTGGCTGAA 15200
      S  M  T  D  P  A  P  Q  D  M  S  S  L  Q  D  A  L  H  D  P  D  L  I  A  W  L  N
15201 TGATAATTCTAAGGATGTGCTGCCTCACGGCCCTGTACCAGCACTGGAGACCATCAATTTGGATCCACCTTCTCCAAGGT 15280
       D  N  S  K  D  V  L  P  H  G  P  V  P  A  L  E  T  I  N  L  D  P  P  S  P  R  Y
15281 ACAGCCCATCCATGCCGGATCTCACCCCCACACGATCGCGGTCAGTGGCAGATACCCCTGCTGCAGACAATGCAGCCCTC 15360
       S  P  S  M  P  D  L  T  P  T  R  S  R  S  V  A  D  T  P  A  A  D  N  A  A  L
15361 CCCTCCCACCAGGCCCCTACTGACCTACTGACCCTCTCCCCTGGTCCTTCTGACTTTGATCTCGAGCTGGCAGCTCCACG 15440
      P  S  H  Q  A  P  T  D  L  L  T  L  S  P  G  P  S  D  F  D  L  E  L  A  A  P  R
15441 CCCCCCATTGTCTGTAACTACAGACACCCCCCATCCTCGGCGTGTGGAAGAAAGCCGCCCCTCTACCCCTATCAAGGCAC 15520
       P  P  L  S  V  T  T  D  T  P  H  P  R  R  V  E  E  S  R  P  S  T  P  I  K  A  R
15521 GGTTGGGCCCGCCAGTTGCAGAAGAGAGCGTTGGGAAACGCTCCCCACCGGGCCCCAGCACCTCTCACCAGAAGAAAGAG 15600
       L  G  P  P  V  A  E  E  S  V  G  K  R  S  P  P  G  P  S  T  S  H  Q  K  K  E
15601 TCCAGCACTGGCAGGACCTCATATGGAGCCGCGGTGGACAGGGCGGTAAAAACCTGGCATCAGCCTAAGGCCGAAGGGTA 15680
      S  S  T  G  R  T  S  Y  G  A  A  V  D  R  A  V  K  T  W  H  Q  P  K  A  E  G  Y
15681 TGACAAAAGATACCGACCAAACAAGATGGGGCCCCCCAAGGCCCACACCTCCTTCACTCGGTCAGGACCCCCTGCACCTC 15760
       D  K  R  Y  R  P  N  K  M  G  P  P  K  A  H  T  S  F  T  R  S  G  P  P  A  P  R
15761 GTCCCCATTACCCACCGTATGTCCCTGCCAAGATCCAACCATTGATCCCCCCTGCTCTTCTTTCTCGGGCTCCTAGGCCT 15840
       P  H  Y  P  P  Y  V  P  A  K  I  Q  P  L  I  P  P  A  L  L  S  R  A  P  R  P
                          3'-LTR
15841 GATTCCAAGGAGAGGGTGTCAAGAGACCCGACAGCTCCGGTCATCACTAGAATTGGAACCAGACCTATGGAGCCTTCACT 15920
      D  S  K  E  R  V  S  R  D  P  T  A  P  V  I  T  R  I  G  T  R  P  M  E  P  S  L
15921 TGACCCCCAGCAGAGGAGGGAGTGTGACAGGAAAAGAAGCCACAGGAAGAATCGAGGTGAAGGCCGAGAAGAGAAGAAAA 16000
       D  P  Q  Q  R  R  E  C  D  R  K  R  S  H  R  K  N  R  G  E  G  R  E  E  K  K  M
                        Acc2 ORF end
16001 TGAGGCGACAGGGAGGAGGTGAAATGATTTGAAGTTAATCAATTTTAATGCTGGCAAAAATACATATGTAACTTGAGTAA 16080
       R  R  Q  G  G  G  E  M  I  *
16081 TGATTATGCTTAATTGCAATGTGAATGATTAACTAATGTACTATGATGATTAATGTGTTTTCAAATGTAAAATGGTGATA 16160
16161 TAGAATAGAAAGAATCAAAGTGTGTCCTAAGATGAAGGAAGGTTGTGTGTGTGTGCTGTTTTGGACGGTGAAAAGTACAG 16240
16241 GGCACATTTCTGGATAATAGGAAAAACAGATGTGTGGGCTTTTCTGGAATGTCCCAGAGGCCCTGTGGTGAAAAACAAAG 16320
16321 TGGTTTTAACTCACCACAGAAAAAGTCCCTAGCAGTCCCATAGCAGAAAGTCAGCAGGATTACCTCATTCTGGAAAGTAC 16400
```

16401 AGAAAAACACATGATGGGAAGTCCCCACCCTTCCACAGAAAGGAAGGAGGGGGGCGAACGAGCCCAGAAAGAGTATAAAA 16480

16481 GGCCAAGCAGTCCAGCAGTAAAGCGTCTTCTGGCCATCGACTGGAGAAGAAGCAGCAAGCAGCGAGGACATCGAGAGCAG 16560

16561 AGGAGCAAGCAGATCCGAAGATCATCTCATCTGGGGCAAGCTGACAGATTCCCCTCCGGCGATCTAAGGACCGAAGACCA 16640

16641 AATTGGACCCAGCCCAACTCTGAGAAGTTCTTTTCAGGCAACAATCTGATGCACCTCATGGGGGTAAAACCGACTGCTCT 16720

16721 AACCTGTCCTGCCGTCTCCAAATTAACAGAAAATGCCACACCACCTAATTAGAAAGATAGGTCAGCATATTAGAGCATTT 16800

16801 TATTTTTATCACCATCAATTATTAATCAATATTCTTAATCTGTATAATCAAATGTGGCTTTTCTTGCAAATTCCTATTAA 16880

16881 ACTGCTTACAGCATCATAAAGACAAATTCCTGACTGGACATTGATTTTTAAAGTTGTCCTTTAAAGTTTTTCTCTGATTA 16960

16961 CATGAGAGTTTTGTTACTGTAAAGCAACAAGGTAATAACATATTAAAGAGTATGGTTGCTTTGAAGCCAAGAGGTAATAA 17040

17041 AATGAGGTCATAGATCACAAATAAGTTCATTTTTAATGGTTATTAACCTGGGGAGATAGGATAACTCCCCTTTAACAGTT 17120

17121 CCCTTGAGACCTATTGTACAGGTCACTAAATCTCATCTCGGCCACAACAAGAAGTGCACATCCTACGAGGGAGCGGCTGC 17200

17201 CAGGAACGATAGTGACCGGCAGATGGAGGCGTCCTGAGCTACTACGCCGACCCAGAGGCCGGGGTTCACGAAGGGAGCGA 17280

17281 CTACCGGAAGGAGGGCAGCTGGAGGTCGAGCTCAAGGCTCCCGCCTGATGCCTCCCGGAGGAGACACGTCAGACTCAGCC 17360

17361 ACACCGGTCACCCCCAGGTGGCTGTAAGTCTGGTCCATCGCCTCCAACCCAAGAGGGAGACATCGTCTCCTCTCCAAGAC 17440

17441 CCACCAGTGTCCGAGTGGGTAAACTGGGGGAGGAGCCACAAAGGATTT**CA**

Supplementary Figure 2 Detailed descriptions of FLERV genomes. Four genomes were annotated in detail, including NviFLERV-1, NviFLERV-2, AciFLERV, and AliFLERV (from top to bottom). Protein open reading frame (ORF) locations were determined by sequence similarity to mammalian foamy viruses and based on the distribution of stop and start codons, determined by Open Reading Frame Finder. Primer binding sites (PBS), polypurine tracts (PPT), and central polypurine tracts (cPPT) are indicated by bold type and solid lines. Long terminal repeats (LTRs), Gag, Pol, Env, Acc1a, Acc1b, and Acc2 protein sequences are highlighted. Several conserved domains and catalytic centres are highlighted in darker colours. Putative insertion and deletion mutations are indicated by red dashes. The beginning and the end of ORFs are indicated by arrows and square-arrows, respectively.

Gag

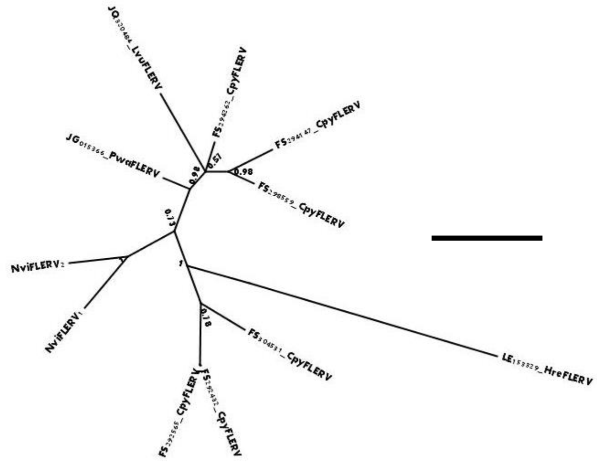

Env

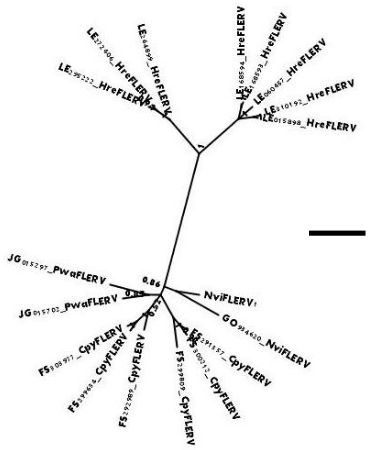

Accessory gene

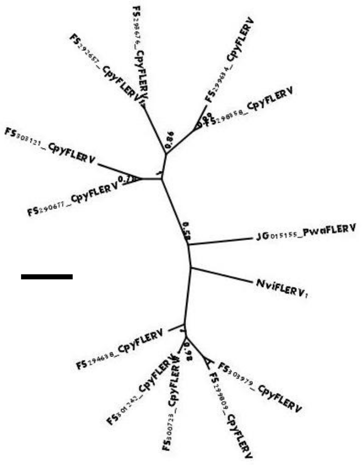

Supplementary Figure 3 Phylogenies of salamander FLERVs. Unrooted Bayesian phylogenies of salamander FLERVs were estimated from Gag (left), Env (right) and accessory protein (middle) alignments. Preceding viral names are the contig accession numbers containing viral sequences used to reconstruct the phylogeny. The scale bars (solid black lines, next to the trees) represent genetic divergence of length 0.6 and are in units of amino-acid substitutions per site. The numbers on nodes are Bayesian posterior probability node support values.

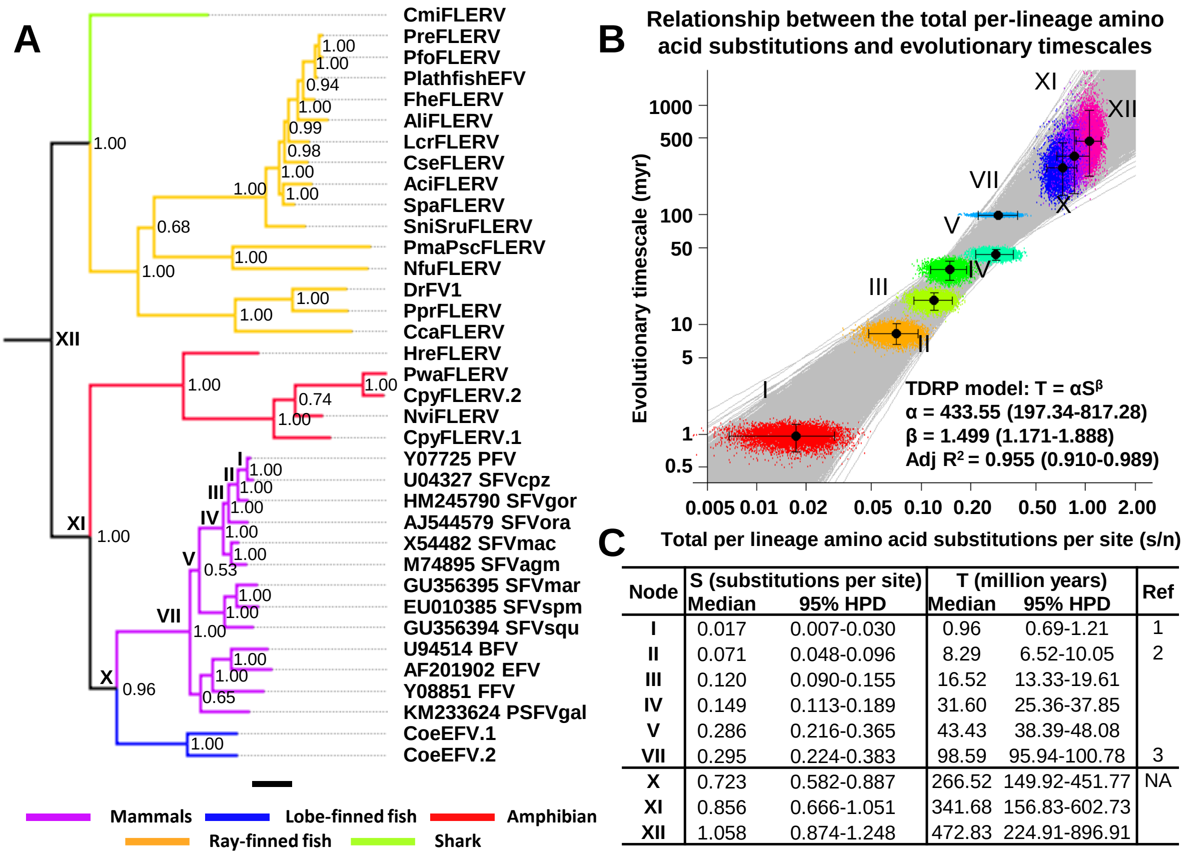

C

Node	S (substitutions per site)		T (million years)		Ref
	Median	95% HPD	Median	95% HPD	
I	0.017	0.007-0.030	0.96	0.69-1.21	1
II	0.071	0.048-0.096	8.29	6.52-10.05	2
III	0.120	0.090-0.155	16.52	13.33-19.61	
IV	0.149	0.113-0.189	31.60	25.36-37.85	
V	0.286	0.216-0.365	43.43	38.39-48.08	
VII	0.295	0.224-0.383	98.59	95.94-100.78	3
X	0.723	0.582-0.887	266.52	149.92-451.77	NA
XI	0.856	0.666-1.051	341.68	156.83-602.73	
XII	1.058	0.874-1.248	472.83	224.91-896.91	

Supplementary Figure 4 Phylogenetic analyses with maximum likelihood ancestral sequences. (**A**) A Bayesian phylogeny of FV and maximum-likelihood ancestral sequences of FLERVs, rooted according to the tree in Fig. 3, left. Preceding FV names are accession numbers. Arabic numerals on nodes are Bayesian posterior probability node support values. Roman numerals indicate the nodes of which the total per-lineage substitutions to the chimpanzee simian FV (U14327 SFVcpz) were used to construct the time-dependent rate phenomenon (TDRP) model (see **B**). The numbers correspond to those in Fig. 3, left. The scale bar (solid black line, underneath the tree) represents genetic divergence of length 0.2 and is in units of amino-acid substitutions per site. (**B**) TDRP model and the timescales of FLERVs. The total per-lineage amino acid substitutions (S) from various nodes to SFVcpz are plotted against corresponding evolutionary timescales (T). The S and T estimates are labelled with Roman numerals, referring to nodes in **A**. Solid black dots are median estimates, and the associated 95% highest probability density (95% HPDs) intervals are indicated by error bars. The T estimates of node I-V, and VII were directly inferred from those of their hosts[1–3] (see **C**). 7,500 power-law TDRP models were fitted to the posterior distributions of the S and T estimates of the nodes I-V, and VII (grey lines). The median model parameter values, adjusted R^2 scores, and corresponding 95% HPDs (in the parentheses) are shown in the bottom right. The model was extrapolated to infer the branching date of lobe-finned fish FLERV lineage (node X), the separation date of salamander FLERV lineage (node XI), and the age of the entire FV/FLERV clade (node XII). (**C**) S estimates and T estimates used in the TDRP model reconstruction (above the line) and the inference of the timescales of FLERVs (below the line). The node numbers refer to those on the viral tree in **A**.

Supplementary Table 1: Foamy virus-like reverse transcriptase nucleotide sequences retrieved from publically available nucleotide databases.

Accession no.	Position*	Species	Class	Databases**
FS296312.1	128-748	*Cynops pyrrhogaster*	Amphibia	est
FS302975.1	c447-1	*Cynops pyrrhogaster*	Amphibia	est
FS304159.1	c407-3	*Cynops pyrrhogaster*	Amphibia	est
FS304221.1	c407-3	*Cynops pyrrhogaster*	Amphibia	est
FS304248.1	c407-3	*Cynops pyrrhogaster*	Amphibia	est
FS304312.1	c407-3	*Cynops pyrrhogaster*	Amphibia	est
FS313726.1	31-578	*Cynops pyrrhogaster*	Amphibia	est
LE148026.1	c4695-4291	*Hynobius retardatus*	Amphibia	TSA
LE148029.1	c2990-2429	*Hynobius retardatus*	Amphibia	TSA
LE271194.1	c707-273	*Hynobius retardatus*	Amphibia	TSA
JF490018.1	c14498-13875	*Notophthalmus viridescens*	Amphibia	HTGS
JF490018.1	c1456-836	*Notophthalmus viridescens*	Amphibia	HTGS
JG014957.1	c583-134	*Pleurodeles waltl*	Amphibia	est
JG015238.1	c753-224	*Pleurodeles waltl*	Amphibia	est
CX312570.1	90-620	*Xenopus tropicalis*	Amphibia	est
XM_007890932.1	329-958	*Callorhinchus milii*	Chondrichthyes	nr
AAVX02007855.1	35070-35699	*Callorhinchus milii*	Chondrichthyes	wgs
AAVX02008076.1	17550-18104	*Callorhinchus milii*	Chondrichthyes	wgs
AAVX02021479.1	c5271-4831	*Callorhinchus milii*	Chondrichthyes	wgs
AAVX02030290.1	18590-19123	*Callorhinchus milii*	Chondrichthyes	wgs
CCOE01000298.1	569-1215	*Amphilophus citrinellus*	Osteichthyes	wgs
CCOE01000352.1	308390-309034	*Amphilophus citrinellus*	Osteichthyes	wgs
CCOE01000398.1	23919-24563	*Amphilophus citrinellus*	Osteichthyes	wgs
CCOE01000548.1	20338-20976	*Amphilophus citrinellus*	Osteichthyes	wgs
CCOE01000821.1	6654-7292	*Amphilophus citrinellus*	Osteichthyes	wgs
CCOE01001074.1	c15937-15281	*Amphilophus citrinellus*	Osteichthyes	wgs
CCOE01001468.1	1422655-1423299	*Amphilophus citrinellus*	Osteichthyes	wgs
CCOE01001819.1	51290-51934	*Amphilophus citrinellus*	Osteichthyes	wgs
CCOE01001827.1	10491-11135	*Amphilophus citrinellus*	Osteichthyes	wgs
CCOE01001950.1	28917-29553	*Amphilophus citrinellus*	Osteichthyes	wgs
CCOE01002087.1	c18362-17724	*Amphilophus citrinellus*	Osteichthyes	wgs
CCOE01002093.1	c11959-11348	*Amphilophus citrinellus*	Osteichthyes	wgs
CCOE01002105.1	c19161-18517	*Amphilophus citrinellus*	Osteichthyes	wgs
CCOE01002121.1	c10246-9602	*Amphilophus citrinellus*	Osteichthyes	wgs
CCOE01002123.1	12470-13081	*Amphilophus citrinellus*	Osteichthyes	wgs
CCOE01002125.1	8124-8764	*Amphilophus citrinellus*	Osteichthyes	wgs
CCOE01002130.1	c12245-11601	*Amphilophus citrinellus*	Osteichthyes	wgs
CCOE01002251.1	2260-2904	*Amphilophus citrinellus*	Osteichthyes	wgs
CCOE01002262.1	4525-5169	*Amphilophus citrinellus*	Osteichthyes	wgs
CCOE01002516.1	c11811-11167	*Amphilophus citrinellus*	Osteichthyes	wgs
CCOE01003376.1	c7539-6895	*Amphilophus citrinellus*	Osteichthyes	wgs
CCOE01004540.1	553-1197	*Amphilophus citrinellus*	Osteichthyes	wgs
AWGY01041462.1	c2004-1375	*Anoplopoma fimbria*	Osteichthyes	wgs
LDAR01045383.1	5236-5890	*Austrofundulus limnaeus*	Osteichthyes	wgs
LDAR01054356.1	c1344-688	*Austrofundulus limnaeus*	Osteichthyes	wgs
LDAR01059718.1	c5355-4702	*Austrofundulus limnaeus*	Osteichthyes	wgs
LDAR01064714.1	4570-4854	*Austrofundulus limnaeus*	Osteichthyes	wgs
LDAR01074257.1	c34558-33913	*Austrofundulus limnaeus*	Osteichthyes	wgs
LDAR01076447.1	c7566-6912	*Austrofundulus limnaeus*	Osteichthyes	wgs
LDAR01080842.1	c3142-2487	*Austrofundulus limnaeus*	Osteichthyes	wgs
LDAR01084167.1	6421-7077	*Austrofundulus limnaeus*	Osteichthyes	wgs

LDAR01086575.1	c462-84	*Austrofundulus limnaeus*	Osteichthyes	wgs
LDAR01088004.1	8364-9064	*Austrofundulus limnaeus*	Osteichthyes	wgs
LDAR01099687.1	c12143-11732	*Austrofundulus limnaeus*	Osteichthyes	wgs
LDAR01103919.1	c10461-9806	*Austrofundulus limnaeus*	Osteichthyes	wgs
LDAR01108616.1	c10961-10305	*Austrofundulus limnaeus*	Osteichthyes	wgs
LDAR01109605.1	906-1562	*Austrofundulus limnaeus*	Osteichthyes	wgs
LDAR01110171.1	5619-6273	*Austrofundulus limnaeus*	Osteichthyes	wgs
LDAR01111596.1	c4939-4289	*Austrofundulus limnaeus*	Osteichthyes	wgs
LDAR01113167.1	2599-3256	*Austrofundulus limnaeus*	Osteichthyes	wgs
LDAR01113985.1	3214-3483	*Austrofundulus limnaeus*	Osteichthyes	wgs
LDAR01116364.1	6233-6883	*Austrofundulus limnaeus*	Osteichthyes	wgs
LDAR01120049.1	9570-10592	*Austrofundulus limnaeus*	Osteichthyes	wgs
LDAR01123043.1	c9570-8914	*Austrofundulus limnaeus*	Osteichthyes	wgs
LDAR01125316.1	605-1262	*Austrofundulus limnaeus*	Osteichthyes	wgs
LDAR01127107.1	995-1645	*Austrofundulus limnaeus*	Osteichthyes	wgs
LDAR01128274.1	10861-11112	*Austrofundulus limnaeus*	Osteichthyes	wgs
LDAR01128742.1	c710-318	*Austrofundulus limnaeus*	Osteichthyes	wgs
LDAR01130590.1	3190-3845	*Austrofundulus limnaeus*	Osteichthyes	wgs
AGRG01008745.1	c6819-6179	*Cynoglossus semilaevis*	Osteichthyes	wgs
AGRG01061695.1	1422-2066	*Cynoglossus semilaevis*	Osteichthyes	wgs
LN590673.1	c14574362-14573703	*Cyprinus carpio*	Osteichthyes	nr
LN590686.1	c11209149-11208547	*Cyprinus carpio*	Osteichthyes	nr
LN590902.1	c92822-92217	*Cyprinus carpio*	Osteichthyes	nr
LN591772.1	78803-79403	*Cyprinus carpio*	Osteichthyes	nr
LN592165.1	c8233-7631	*Cyprinus carpio*	Osteichthyes	nr
LN594197.1	561216-561659	*Cyprinus carpio*	Osteichthyes	nr
LN594197.1	581455-582057	*Cyprinus carpio*	Osteichthyes	nr
CAAK05043237.1	c30960-30547	*Danio rerio*	Osteichthyes	wgs
CAAK05053864.1	c19938-19492	*Danio rerio*	Osteichthyes	wgs
CABZ01012381.1	c41144-40698	*Danio rerio*	Osteichthyes	wgs
CABZ01026504.1	c11500-11186	*Danio rerio*	Osteichthyes	wgs
CABZ01041340.1	c31043-30630	*Danio rerio*	Osteichthyes	wgs
CABZ01054182.1	53870-54316	*Danio rerio*	Osteichthyes	wgs
CABZ01059307.1	c8345-7899	*Danio rerio*	Osteichthyes	wgs
CABZ01098205.1	c3177-2731	*Danio rerio*	Osteichthyes	wgs
CZQB01082620.1	c1695-1249	*Danio rerio*	Osteichthyes	wgs
CZQB01127375.1	c17284-16871	*Danio rerio*	Osteichthyes	wgs
CZQB01170393.1	314-640	*Danio rerio*	Osteichthyes	wgs
LKPD01020285.1	c16071-15658	*Danio rerio*	Osteichthyes	wgs
CBXY010016181.1	55744-55998	*Dicentrarchus labrax*	Osteichthyes	wgs
AZJR02000232.1	282450-283051	*Esox lucius*	Osteichthyes	wgs
JXMV01012181.1	c3197-2559	*Fundulus heteroclitus*	Osteichthyes	wgs
JXMV01100753.1	9176-9808	*Fundulus heteroclitus*	Osteichthyes	wgs
CAEA01131311.1	6-536	*Gadus morhua*	Osteichthyes	wgs
CAEA01539013.1	c3858-3220	*Gadus morhua*	Osteichthyes	wgs
JPYK01043025.1	c1753-1184	*Larimichthys crocea*	Osteichthyes	wgs
JRPU01006056.1	c71664-71026	*Larimichthys crocea*	Osteichthyes	wgs
JRPU01007253.1	7552-8190	*Larimichthys crocea*	Osteichthyes	wgs
JRPU01008437.1	c27096-26458	*Larimichthys crocea*	Osteichthyes	wgs
JRPU01010616.1	45179-45817	*Larimichthys crocea*	Osteichthyes	wgs
JRPU01011225.1	4195-4833	*Larimichthys crocea*	Osteichthyes	wgs
JRPU01012463.1	c2936-2298	*Larimichthys crocea*	Osteichthyes	wgs
JRPU01013645.1	c5778-5140	*Larimichthys crocea*	Osteichthyes	wgs
JRPU01018720.1	c9347-8778	*Larimichthys crocea*	Osteichthyes	wgs

JRPU01021077.1	c9543-8905	*Larimichthys crocea*	Osteichthyes	wgs
LBLR01010097.1	c8752-8234	*Lates calcarifer*	Osteichthyes	wgs
GAIB01031349.1	757-1296	*Nothobranchius furzeri*	Osteichthyes	TSA
ABLO01001532.1	c656-33	*Nothobranchius furzeri*	Osteichthyes	wgs
ACCZ01000769.1	37-660	*Nothobranchius furzeri*	Osteichthyes	wgs
JNBZ01063262.1	3578-4201	*Nothobranchius furzeri*	Osteichthyes	wgs
JNBZ01067388.1	c1941-1342	*Nothobranchius furzeri*	Osteichthyes	wgs
JNBZ01070977.1	205-828	*Nothobranchius furzeri*	Osteichthyes	wgs
JNBZ01105729.1	807-1430	*Nothobranchius furzeri*	Osteichthyes	wgs
JNBZ01114434.1	c1351-1037	*Nothobranchius furzeri*	Osteichthyes	wgs
JNBZ01114434.1	c885-262	*Nothobranchius furzeri*	Osteichthyes	wgs
JNBZ01238562.1	c630-7	*Nothobranchius furzeri*	Osteichthyes	wgs
AERX01018483.2	c31806-31169	*Oreochromis niloticus*	Osteichthyes	wgs
JACM01000693.1	1447-1923	*Periophthalmodon schlosseri*	Osteichthyes	wgs
JACM01004568.1	c8464-7963	*Periophthalmodon schlosseri*	Osteichthyes	wgs
JACM01028065.1	c506-3	*Periophthalmodon schlosseri*	Osteichthyes	wgs
JACL01052273.1	c2223-1576	*Periophthalmus magnuspinnatus*	Osteichthyes	wgs
JNCD01010179.1	2919-3554	*Pimephales promelas*	Osteichthyes	wgs
JNCD01019993.1	49258-49905	*Pimephales promelas*	Osteichthyes	wgs
JNCD01029789.1	3921-4561	*Pimephales promelas*	Osteichthyes	wgs
JNCD01073002.1	c7341-6694	*Pimephales promelas*	Osteichthyes	wgs
JNCE01099165.1	4812-5452	*Pimephales promelas*	Osteichthyes	wgs
JNCE01145994.1	7578-8225	*Pimephales promelas*	Osteichthyes	wgs
JNCE01342880.1	3061-3708	*Pimephales promelas*	Osteichthyes	wgs
JNCE01566843.1	c497-3	*Pimephales promelas*	Osteichthyes	wgs
AYCK01020712.1	11627-12271	*Poecilia formosa*	Osteichthyes	wgs
AYCK01021113.1	4374-5018	*Poecilia formosa*	Osteichthyes	wgs
AYCK01021113.1	6552-7196	*Poecilia formosa*	Osteichthyes	wgs
AYCK01023761.1	c12032-11373	*Poecilia formosa*	Osteichthyes	wgs
AYCK01024933.1	c1579-935	*Poecilia formosa*	Osteichthyes	wgs
AYCK01026224.1	c13615-12971	*Poecilia formosa*	Osteichthyes	wgs
AYCK01027102.1	c10638-9994	*Poecilia formosa*	Osteichthyes	wgs
AYCK01029392.1	4062-4705	*Poecilia formosa*	Osteichthyes	wgs
AZHG01008165.1	c9444-8800	*Poecilia reticulata*	Osteichthyes	wgs
AZHG01026008.1	c1689-1071	*Poecilia reticulata*	Osteichthyes	wgs
AZHG01027173.1	4486-5013	*Poecilia reticulata*	Osteichthyes	wgs
AZHG01028727.1	c16211-15567	*Poecilia reticulata*	Osteichthyes	wgs
AZHG01031876.1	3998-4642	*Poecilia reticulata*	Osteichthyes	wgs
AZHG01037968.1	c638-3	*Poecilia reticulata*	Osteichthyes	wgs
AUPR01019601.1	4426-5079	*Sebastes nigrocinctus*	Osteichthyes	wgs
AUPQ01030678.1	c8375-7722	*Sebastes rubrivinctus*	Osteichthyes	wgs
JMKM01038484.1	c2396-1752	*Stegastes partitus*	Osteichthyes	wgs
JMKM01039462.1	c1764-1129	*Stegastes partitus*	Osteichthyes	wgs
JMKM01039980.1	8626-9270	*Stegastes partitus*	Osteichthyes	wgs
BADN01112239.1	502-1138	*Thunnus orientalis*	Osteichthyes	wgs
BADN01125085.1	882-1301	*Thunnus orientalis*	Osteichthyes	wgs
AGAJ01031885.1	2681-3136	*Xiphophorus maculatus*	Osteichthyes	wgs
AGAJ01039643.1	21494-21946	*Xiphophorus maculatus*	Osteichthyes	wgs
AGAJ01041163.1	c9244-8789	*Xiphophorus maculatus*	Osteichthyes	wgs
AGAJ01045326.1	c9270-8815	*Xiphophorus maculatus*	Osteichthyes	wgs
AGAJ01045559.1	4290-4745	*Xiphophorus maculatus*	Osteichthyes	wgs
AGAJ01047098.1	715-1170	*Xiphophorus maculatus*	Osteichthyes	wgs
AGAJ01049791.1	c1128-673	*Xiphophorus maculatus*	Osteichthyes	wgs
AGAJ01050163.1	c1105-650	*Xiphophorus maculatus*	Osteichthyes	wgs

AGAJ01050296.1	4013-4468	*Xiphophorus maculatus*	Osteichthyes	wgs
AGAJ01052256.1	247-702	*Xiphophorus maculatus*	Osteichthyes	wgs
AGAJ01053413.1	281-736	*Xiphophorus maculatus*	Osteichthyes	wgs
GAPS01007249.1	313-729	*Latimeria menadoensis*	Sarcopterygii	TSA
AFYH01036414.1	c700-254	*Latimeria chalumnae*	Sarcopterygii	wgs
AFYH01178313.1	c4935-4489	*Latimeria chalumnae*	Sarcopterygii	wgs
AFYH01184753.1	c1256-807	*Latimeria chalumnae*	Sarcopterygii	wgs
AFYH01210236.1	8698-9144	*Latimeria chalumnae*	Sarcopterygii	wgs
AFYH01247568.1	c3914-3468	*Latimeria chalumnae*	Sarcopterygii	wgs
BAHO01057271.1	c5029-4586	*Latimeria chalumnae*	Sarcopterygii	wgs
BAHO01073407.1	1167-1613	*Latimeria chalumnae*	Sarcopterygii	wgs
BAHO01074903.1	c5564-5118	*Latimeria chalumnae*	Sarcopterygii	wgs
BAHO01088137.1	c4040-3594	*Latimeria chalumnae*	Sarcopterygii	wgs
BAHO01107246.1	c7021-6575	*Latimeria chalumnae*	Sarcopterygii	wgs
BAHO01122095.1	c1008-562	*Latimeria chalumnae*	Sarcopterygii	wgs
BAHO01123364.1	c6228-5782	*Latimeria chalumnae*	Sarcopterygii	wgs
BAHO01124408.1	2691-3137	*Latimeria chalumnae*	Sarcopterygii	wgs
BAHO01126659.1	7079-7525	*Latimeria chalumnae*	Sarcopterygii	wgs
BAHO01131885.1	c1942-1496	*Latimeria chalumnae*	Sarcopterygii	wgs
BAHO01162318.1	8104-8550	*Latimeria chalumnae*	Sarcopterygii	wgs
BAHO01174129.1	2133-2579	*Latimeria chalumnae*	Sarcopterygii	wgs
BAHO01177875.1	19240-19686	*Latimeria chalumnae*	Sarcopterygii	wgs
BAHO01190208.1	c22322-21873	*Latimeria chalumnae*	Sarcopterygii	wgs
BAHO01198287.1	c7041-6595	*Latimeria chalumnae*	Sarcopterygii	wgs
BAHO01243374.1	c604-158	*Latimeria chalumnae*	Sarcopterygii	wgs
BAHO01244829.1	c1902-1456	*Latimeria chalumnae*	Sarcopterygii	wgs
BAHO01246974.1	7936-8379	*Latimeria chalumnae*	Sarcopterygii	wgs
BAHO01312704.1	c3294-2848	*Latimeria chalumnae*	Sarcopterygii	wgs
BAHO01321068.1	23843-24289	*Latimeria chalumnae*	Sarcopterygii	wgs
BAHO01335329.1	c774-328	*Latimeria chalumnae*	Sarcopterygii	wgs
BAHO01387613.1	9178-9624	*Latimeria chalumnae*	Sarcopterygii	wgs
BAHO01391119.1	23461-23907	*Latimeria chalumnae*	Sarcopterygii	wgs
BAHO01395088.1	c2134-1688	*Latimeria chalumnae*	Sarcopterygii	wgs
BAHO01407282.1	c2606-2160	*Latimeria chalumnae*	Sarcopterygii	wgs

*the letter ‘c’ indicates complementary sequences.

** ‘nr’ = non-redundant nucleotide sequences; ‘est’ = expressed sequence tags; ‘HTGS’ = high throughput genomic sequences; ‘wgs’ = whole genome shotgun sequences; and ‘TSA’ = transcriptome shotgun assembly sequences.

Supplementary Note 1 Detailed genomic annotations of FLERVs

Four genomes were annotated in detail, including NviFLERV-1, NviFLERV-2, AciFLERV, and AliFLERV.

NviFLERV-1

NviFLERV-1 is a full-length endogenous retroviral element (9,200 nucleotides (nt)). It was found in the genome of eastern newt (*Notophthalmus viridescens*) on the contig JF490018, locating at the nt position c17,626 to c8,434. (The nt positions are with respect to the contig length; the letter 'c' indicates that the element is characterised on the complementary sequence.) By blasting NviFLERV-1 against itself, we were able to locate its 5'- and 3'-long terminal repeat (LTR) to be at the nt position c17,626-16,667 (960 nt) and c9,407-8,434 (974 nt), respectively. Target site duplications (TSDs) were also found (5'-GAGT-3'), flanking NviFLERV-1. Situated on the 3' end of the 5'-LTR is a $tRNA^{Asn}$-utilising putative primer binding site (PBS), identified via sequence homology to be at the nt position c16,673-16,692 (TACCCAACGTGG). This is similar to the PBS utilised by prosimian galago foamy virus, but differs from those of other mammalian foamy viruses (FVs), which are $tRNA^{Lys}$-utilising PBSs. The putative *gag*, *pol*, and *env* genes were predicted to be at the nt position c16,368-14,859 (1,510 nt), c14,983-11,458 (3,526 nt), c11,467-10,041 (1,427 nt), respectively, identified by sequence similarity to those of simian FVs (SFVs). Several in-frame stop codons and frameshift mutations were found, indicative of the defective nature of NviFLERV-1, typical for an ERV. We could also locate (defective) polypurine tracts (PPT) for the dual initiation of plus-stand DNA synthesis to be on the 5' boundary of the 3'-LTR (GATCTTAGGGGGG; nt c9,420-9,408) and at the centre of the genome toward the 3' terminus of *pol* gene (GAGTCCCTGGGGG; nt c12,103-12,091) as anticipated. The putative Gag (503 aa), Pol (1,176 aa), and Env (475 aa) proteins are most similar to the Gag (BLASTp best hit: AIM40342; $E = 1 \times 10^{-14}$), Pol (BLASTp best hit: ABV59399; $E = 2 \times 10^{-130}$), and Env (BLASTp best hit: AKM21186; $E = 4 \times 10^{-8}$) proteins of SFVs, confirming that it is FV-like. We however noted that the predicted Env protein of NviFLERV-1 (475 aa) is much shorter than those of mammalian FVs (~985 aa). Sequence investigation revealed that the N-terminus was missing. This could be due to host genomic deletion or a genuine absence of the sequence. Exogenous viral counterparts are needed for further examination. Within the Pol putative protein, we were able to locate the reverse transcriptase (aa 163-360; the amino acid positions are with respect to the length of the putative Pol protein), RNaseH (aa 594-739), and integrase core domain (aa 877-988). An aspartic protease domain, which exists in all mammalian FV Pol, could not be found however. Between the *env* and 3'-LTR is a stretch of uniquely-mapping 492 nt (nt c10,048-9,557). BLASTx showed that it exhibits some similarity to a gene of Porcine reproductive and respiratory syndrome virus (BLASTx: AEQ61854; $E = 6 \times 10^{-4}$). In mammalian FVs, this region contains accessory genes (*bel1* and *bel2*). Given its position and the fact that it exhibits some similarity to a viral gene, we believe that it is likely an accessory gene. We in turn designated it an *'acc'* gene. To examine whether or not it is indeed an accessory gene, exogenous viral counterparts are required.

NviFLERV-2

NviFLERV-2 is a truncated element (4,391 nt), discovered in the genome of eastern newt (*N. viridescens*) on the some contig that NviFLERV-1 was found (JF490018). For NviFLERV-2, we identified the following features via sequence homology; i) a $tRNA^{Asn}$-utilising PBS (TGGCGCCCCAA; nt c3,734-3,714) similar to that of NviFLERV-1, ii) a complete putative *gag* gene (nt c3,401-1,821, 1,581 nt), and iii) a partial *pol* gene (nt c1,945-1, 1,945 nt). By using the LTR of NviFLERV-1 as a probe, we were able to locate the (5' truncated) 5'-LTR of NviFLERV-2 to be at the nt position c4,391-3,735 (656 nt). Similar to those of NviFLERV-1, the putative Gag (526 aa) and Pol (648 aa) proteins of NviFLERV-2 are most similar to the Gag (BLASTp best hit: ADE05994, $E = 2 \times 10^{-18}$) and Pol (BLASTp best hit: ABV59399, $E = 1 \times 10^{-98}$) of SFVs, respectively. Within the putative Pol protein, we located the reverse transcriptase, and (partial) RNaseH domain to be at aa 188-361, and 592-647,

respectively. In contrast to NviFLERV-1, NviFLERV-2 *gag* and *pol* genes do not seem to contain any in-frame stop codon or frameshift mutations.

AciFLERV

9 elements of AciFLERVs were found in the genome of midas cichlid (*Amphilophus citrinellus*), allowing us to construct their ancestral sequence for better genomic annotation. The original elements contain numerous in-frame stop codon and frameshift mutations as well as transposable elements and large insertion-deletion mutations, strongly supporting that they are indeed ERVs. The maximum-likelihood ancestral sequence of AciFLERV is 17,409 nt long, much longer than the length of mammalian FVs ~10kb. By investigating the distribution of start and stop codons, we determined AciFLERV to have 8 open reading frames (ORFs; ORF-1 to -8, from the 5' end of the genome). Again, this is also very different from the typical genomic structure of mammalian FVs, which has only 5 ORFs, including (from the 5' end) *gag*, *pol*, and *env* genes followed by two accessory genes *bel1*, and *bel2*.

We found that the LTRs of AciFLERV are 1,498 nt long (5'-LTR: nt 1-1,498; 3'-LTR: nt 15,912-17,409), and for those with paired LTRs, we were able to identify their TSDs to be 5'-GAAG-3' (CCOE01001074), 5'-GTGT-3' (CCOE01000352), 5'-CAGC-3' (CCOE01001468), and 5'-CTGG-3' (CCOE01000548). On the 3' end of the 5'-LTR is a $tRNA^{Lys}$-utilising PBS (TGGCGCCCAACGTGGGGC; nt 1,501-1,518), similar to those utilised by most mammalian FVs. Our analyses revealed that none of the putative proteins translated from the 8 ORFs exhibit strong similarities to known viral proteins, except for the one derived from ORF-2 (nt 5,220-8,432, 3,213 nt), showing a strong similarity to the Pol protein of equine FV (BLASTp best hit: NP054716; $E = 6 \times 10^{-33}$). We thus designated ORF-2 a *pol* gene. The putative Pol protein contains the aspartic protease (aa 122-193), reverse transcriptase (aa 208-385), RNaseH (aa 608-743), and integrase (aa 839-952) core domain, as is typical of a FV Pol protein. Based on our knowledge of FV genomic structure, we believe that ORF-1 (nt 1,879-5,220, 3,342 nt), which locates on the 5' end of the *pol* gene, is likely a *gag* gene, and together with manual sequence inspection, that ORF-5 (nt 10,889-13,222, 2,334 nt) is likely an *env* gene. Indeed, we observed a weak, but detectable, similarity between the C terminus of the putative protein derived from ORF-5 and SFV Env proteins (BLASTp best hit: AAA47801; E = 0.94), supporting the *env* gene annotation. According to the typical genomic structure of FVs, this in turn makes ORF-6 (nt 13,252-14,010, 759 nt), ORF-7 (nt 14,249-15,001, 753 nt), and ORF-8 (nt 15,173-15,874, 702 nt) likely accessory genes. Further inspection revealed that ORF-6 and ORF-7 proteins are highly similar to one another (BLASTp: $E = 1 \times 10^{-26}$), strongly indicating that they are paralogous. We thus annotated ORF-6, -7, and -8 an *'acc1a'* gene, an *'acc1b'* gene, and an *'acc2'* gene, respectively.

Regarding ORF-3 (nt 8,454-9,848, 1,395 nt) and ORF-4 (nt 9,824-10,825, 1,002 nt), since their proteins do not exhibit strong similarity to any known viral proteins, and their positions do not correspond to any known FV genes, it is still unclear what they are. It is also possible that they might actually be parts of the *pol* and *env* genes, but falsely annotated as separate individual ORFs due to errors in the ancestral sequence reconstruction. However, since the lengths of the predicted Pol and Env proteins of AciFLERV (1,070 and 777 aa, respectively) are already comparable to those of mammalian FVs (~1,150 aa, and ~985 aa, respectively), we believe that this is unlikely. Additionally, we found that contigs that contain these 2 ORFs tend to also contain *gag*, *pol* and/or *env* genes, strongly supporting that they indeed have viral origins, likely existing in the progenitor of AciFLERV. Furthermore, although mammalian FVs do not possess accessory genes between *pol* and *env* genes, other retroviruses do. For example, the lentiviral human immunodeficiency virus contains three accessory genes in this region, namely *vif*, *vpr*, and *vpu*, which are essential for the viral replication, assembly, and release. We thus believe that ORF-3 and ORF-4 are likely viral accessory genes, and designated them '*acc3*' and '*acc4*', respectively.

AliFLERV

23 AliFLERVs were found in the genome of annual killifish (*Austrofundulus limnaeus*), allowing their ancestral sequence to be reconstructed for better genomic annotation. Similar to AciFLERVs, the original elements contain numerous in-frame stop codon and frameshift mutations as well as transposable elements and large insertion-deletion mutations, strongly supporting that they are ERVs. The inferred ancestral sequence of AliFLERV is 17,490 nt long, and by inspecting the distribution of start and stop codons, we found that AliFLERV has 6 ORFs. Again, similar to AciFLERV, AliFLERV is much longer than typical mammalian FVs, and its genomic structure is also very different from those of mammalian FVs.

By BLASTing the sequence against itself, we determined its LTRs to be 1,634 nt long (5'-LTR: nt 1-1,634; 3'-LTR: nt 15,857-17,490), slightly longer than those of AciFLERV. For one of the two elements that has paired LTRs, we found its TSDs to be 5'-ATA[C|A]-3' (LDAR01108616). The TSDs of the other element could not be identified as the contig that harbours the element (LDAR01045383) does not contain complete LTRs (i.e. the 5' end of the 5' LTR and the 3' end of the 3' LTR are missing). A $tRNA^{Lys}$-utilising PBS (TGGTGCCCAACGTGGGGC; nt 1,635-1,652) was identified after the 5'-LTR as anticipated via sequence homology, similar to those of most mammalian FVs and AciFLERV. As observed in the case of AciFLERV, none of the putative proteins derived from the 6 ORFs exhibit strong similarities to known viral proteins, except for the one derived from ORF-2 (nt 5,188-8,403, 3,216 nt), showing a strong similarity to the Pol protein of equine FV (BLASTp best hit: NP054716; $E = 6 \times 10^{-97}$). Hence, we designated AliFLERV ORF-2 a *pol* gene. The putative Pol protein of AliFLERV is highly similar to that of AciFLERV (BLASTp: E = 0, query cover = 99%), containing the protease (aa 120-193), reverse transcriptase (aa 206-383), RNaseH (aa 609-749), and integrase (aa 837-954) core domain. To annotate the rest of the genome, we transferred the gene annotation from AciFLERV to AliFLERV via protein sequence similarity. Our analyses suggest that ORF-1 (nt 1,955-5,191, 3,237 nt), ORF-4 (nt 10,120-12,501, 2,382 nt), and ORF-5 (nt 13,508-14,488, 981 nt) are *gag* gene (BLASTp: $E < 3 \times 10^{-113}$, query cover > 87%), *env* gene (BLASTp: $E = 2 \times 10^{-107}$, query cover = 94%), and *acc1* gene (Acc1a BLASTp: $E = 9 \times 10^{-18}$, query cover = 81%; Acc1b BLASTp: $E = 2 \times 10^{-12}$, query cover = 85%), respectively. Although we could not detect a significant similarity between AciFLERV *acc2* gene and AliFLERV ORF-6, we believe that AliFLERV ORF-6 is likely a *acc2* gene according to its position. Finally, our analyses mapped AciFLERV *acc3* to AliFLERV ORF-3 with a high confidence (nt 8,454-9,848; BLASTp: $E = 8 \times 10^{-113}$, query cover = 90%), and the lengths of the two ORFs are also highly similar (AciFLERV ORF-3: 1,395 nt; AliFLERV ORF-3: 1,424 nt). We therefore annotated ORF-3 as an *acc3* gene.

Supplementary references

1. Stone, A. C. *et al.* More reliable estimates of divergence times in Pan using complete mtDNA sequences and accounting for population structure. *Philos. Trans. R. Soc. Lond. B. Biol. Sci.* **365,** 3277–3288 (2010).
2. Perelman, P. *et al.* A molecular phylogeny of living primates. *PLoS Genet.* **7,** e1001342 (2011).
3. Bininda-Emonds, O. R. P. *et al.* The delayed rise of present-day mammals. *Nature* **446,** 507–512 (2007).
